# Identifying Kinase Substrates via a Heavy ATP Kinase Assay and Quantitative Mass Spectrometry

**DOI:** 10.1038/srep28107

**Published:** 2016-06-27

**Authors:** André C. Müller, Roberto Giambruno, Juliane Weißer, Peter Májek, Alexandre Hofer, Johannes W. Bigenzahn, Giulio Superti-Furga, Henning J. Jessen, Keiryn L. Bennett

**Affiliations:** 1 CeMM Research Center for Molecular Medicine of the Austrian Academy of Sciences, Vienna, Austria; 2 University of Zurich, Department of Chemistry, Zurich, Switzerland

## Abstract

Mass spectrometry-based *in vitro* kinase screens play an essential role in the discovery of kinase substrates, however, many suffer from biological and technical noise or necessitate genetically-altered enzyme-cofactor systems. We describe a method that combines stable γ-[^18^O_2_]-ATP with classical *in vitro* kinase assays within a contemporary quantitative proteomic workflow. Our approach improved detection of known substrates of the non-receptor tyrosine kinase ABL1; and identified potential, new *in vitro* substrates.

Tyrosine kinases play a central role in numerous cellular signal transduction pathways and are frequently found de-regulated in human pathologies^1^,^2^. Ideally, to fully comprehend the function of a kinase; both the interactors and substrates should be known. In recent years, various combinations of *in vitro* kinase assays coupled to mass spectrometry (MS) have emerged to detect kinase substrates^3^. Despite adaptation of the original Kestrel methodology^4^,^5^, non-specific background phosphorylation and quantitation-related noise has limited the sensitivity and reliability of MS-based kinase assays. In an attempt to address the former, Shah *et al*. created adenosine triphosphate (ATP)-analogue-sensitive (AS) kinases that preferentially bind ‘bulky’ ATP derivatives^6^. These engineered AS-kinases specifically mark substrates with, *e.g.*, thiophosphate that can be distinguished from a conventional phosphate moiety by MS. Not all kinases, however, can be converted into AS-kinases and some can lose functionality and/or substrate specificity^7^. A more physiological approach is to utilise naturally-expressed kinases combined with stable isotopically-labelled ATP. Replacement of ^16^O atoms in the γ-phosphate group of ATP with ^18^O isotopes gives a mass shift of 2 a.m.u. per oxygen atom^8^. The kinase-mediated transfer of a ‘heavy’ ^18^O-labelled γ-phosphate group to a protein substrate subsequently marks newly-introduced phospho-moieties^9^,^10^. The advantages of such ATP analogues have been previously demonstrated, *e.g.*, detection of phosphorylation sites^9^; determining specificities of kinase inhibitors^8^; measuring kinase activity *in nucleo*^11^ and identifying CDC28 or ERK1 substrates^10^,^12^. To date, however, the use of ^18^O-labelled ATP in the field of proteomics is not extensive.

We present a ‘heavy’ ATP kinase assay combined with quantitative mass spectrometry (HAKA-MS) that reliably identifies *in vitro* kinase substrates. The well-studied non-receptor tyrosine kinase ABL1 was chosen as a model. The technique enables dissection of *in vitro* substrates from the basal phosphoproteome which is a major challenge with naturally-occurring ‘light’ ATP^3^. This was addressed in HAKA-MS by: (i) global and irreversible inactivation of endogenous kinases using 5′-[p-(fluorosulfonyl)benzoyl]adenosine (FSBA)^13^; (ii) comparison of substrate phosphorylation between the constitutively-active ABL1-PP (P242E, P249E) and the catalytically-inactive ABL1-Kin^−^ (K290R); (iii) visualisation of temporal phosphorylation patterns; and (iv) substitution of naturally-occurring ATP with an ^18^O-labelled version in the *in vitro* kinase assay^9^,^10^,^12^.

## Results

### HAKA-MS Strategy for the Identification of ABL1 substrates

**Figure 1:**
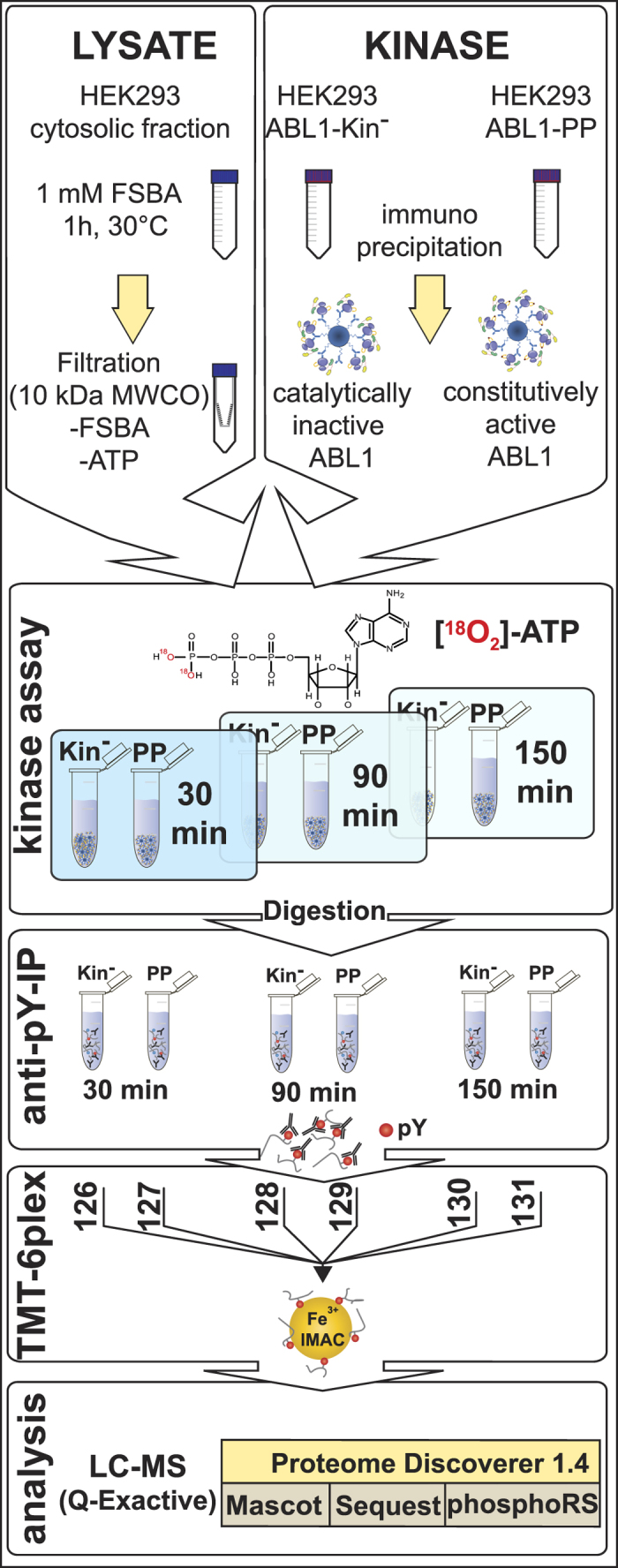
Schematic workflow of HAKA-MS. Cytosolic fractions were treated with the pan-kinase inhibitor FSBA to irreversibly abolish cellular kinase activity. Simultaneous exchange to the kinase assay buffer; and removal of excess FSBA and endogenous ATP was achieved by ultrafiltration. In parallel, constitutively-active ABL1-PP and inactive ABL1-Kin^−^ were immunopurified via protein G sepharose beads. Prior to the kinase assay, treated lysate and washed kinase-bead fractions were equally divided, mixed and the kinase reaction initiated with ‘heavy’ ^18^O-labelled ATP. To obtain the phosphorylation kinetic series, pairs of active/inactive kinase reactions were quenched at defined time points. Following enzymatic digestion, peptides were concentrated with solid-phase extraction and phosphotyrosine-containing peptides enriched via immunoprecipitation. Eluted peptides were chemically-modified with isobaric amine-reactive tandem mass tag (TMT) reagents to multiplex 3 × 2 samples. After a second phosphopeptide enrichment with immobilised metal-affinity chromatography (IMAC), the sample was analysed by LCMS.

**Figure 2:**
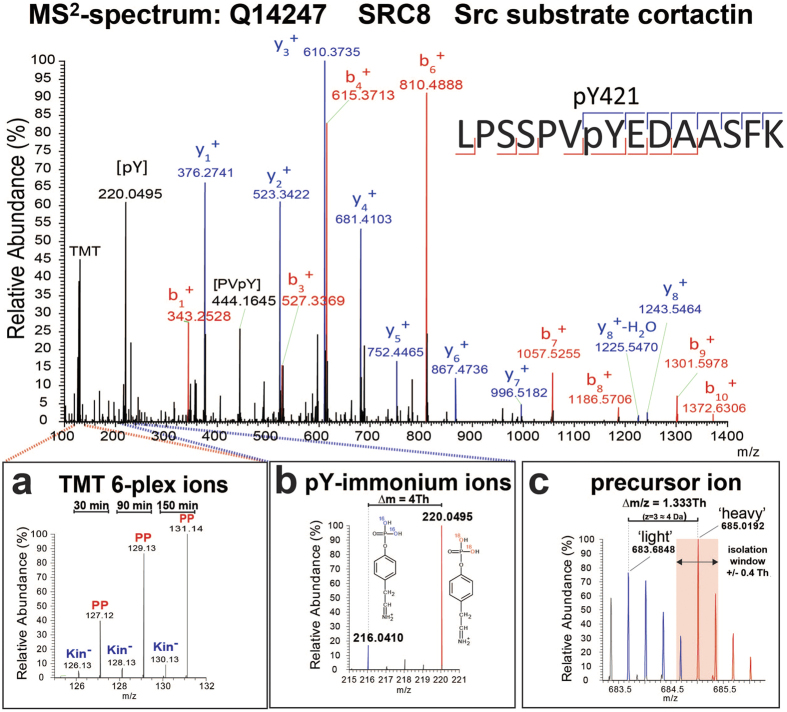
Spectral features of HAKA-MS. An example of an MS^2^ spectrum corresponding to a tryptic peptide derived from SRC8 containing a reported ABL1 phosphorylation site. The assigned fragment ions provide peptide-specific sequence information that is used to computationally match the data to the corresponding peptide in a protein database. Certain fragment ions (*i.e.*, y_8_ and b_7_) unambiguously assign the phosphate group (+84 Da) to Y421 and not to S417, S418 or S426. (**a**) Magnification of the TMT reporter ion region to illustrate the relative abundance of the individual multiplexed samples for this specific phosphotyrosine-containing peptide. TMT 6-plex ion intensities were used to generate individual relative ratios or to plot reaction progress kinetics. (**b**) pY-immonium ions for ‘light’ (*m/z* 216.04) and ‘heavy’ (*m/z* 220.04) versions of the phosphopeptide. The existence and relative abundances of the immonium ions provide unequivocal evidence of tyrosine residue phosphorylation and quality control of the respective spectrum. (**c**) MS^1^ precursor ion of the phosphotyrosine-containing peptide with both the ‘light’ (*m/z* 683.6848) and ‘heavy’ (*m/z* 685.0192) isotopologues. Both isotopologues (blue and red peaks) display a typical ^12^C/^13^C-isotopic peak distribution (1 a.m.u. separation) but with a slight overlap due to the small ∆*m/z* of 1.333 Th. Precursor ion selection of the monoisotopic peak (pink area) was within the isolation window of ±0.4 Th and partially including the ‘light’ isotopologue (fourth ^13^C-isotopic peak).

Cellular immunopurified ABL1-PP and ABL1-Kin^−^ were mixed with an FSBA-treated cytosolic extract of HEK293 cells as illustrated in Fig. 1. FSBA has been widely-used as a prototypic nucleotide-based affinity label for kinases and other ATP-binding proteins. The fluorosulfonyl moiety of this ATP analogue can react with nucleophilic residues, *i.e.*, a conserved lysine in the ATP-binding site of kinases to form a covalent adduct that irreversibly occupies the ATP binding site^14^,^15^. The inhibitory efficiency of FSBA has been previously demonstrated^13^,^14^,^16^ and 1 mM FSBA is sufficient to quench endogenous kinase activity (Supplementary Fig. 1). Any residual kinase activity in the extract will be evident in the kinase control ABL-Kin^−^. Excess FSBA and endogenous ATP were removed via filtration. The kinase reaction was initiated by adding ‘heavy’ ATP and kinase reaction pairs were quenched at 30, 90 and 150 min. Time points were selected based on α-phosphotyrosine (α-pY) immunoblots that showed a temporal increase in phosphorylation for active ABL1-PP before plateauing at 150 minutes (Supplementary Fig. 2). Proteins were reduced, alkylated, digested and phosphotyrosine peptides enriched by immunopurification. After labelling with TMT 6-plex reagents, to enable relative quantitation across the 6 samples (Fig. 2a); pooled samples were further enriched with immobilised metal affinity chromatography (IMAC)^17^ and analysed by nano-LCMS. TMT labelling and peptide pooling was performed after pY-peptide enrichment as an unrealistic amount of the reagent would be required to label milligrams of starting material (*i.e.*, 2–5 mg). To avoid additional experimental variability, it is also critical across the different TMT channels to perform the immunoprecipitation procedure as consistently and carefully as possible. Acquired data were searched against the human SwissProt protein database with serine, threonine (+80 Da) and tyrosine phosphorylation (+80 and +84 Da for ‘light’ and ‘heavy’ versions, respectively) as variable modifications.

HAKA-MS of ABL1 identified 180 unique phosphotyrosine (pY) sites from 130 phosphoproteins that passed the filter criteria and manual validation. Most of the phosphopeptides had a singly-phosphorylated tyrosine residue. Additional phospho-groups at serine, threonine or tyrosine residues were evident in 21 peptides. Matched pY-containing peptides segregated into two distinct groups; containing either ‘light’ or ‘heavy’ phosphotyrosine moieties. Only six pY-peptides were identified as both forms. This was due to: (i) exclusive occupancy of the phosphotyrosine peptide; and/or (ii) co-existence of isotopologues (Fig. 2c) and preferential isolation and fragmentation of the more intense ion. As shown previously^9^, mass increments marked sites of *de novo* phosphorylation and enabled dissection of new phospho-sites from the pre-existing ‘light’ phosphoproteome. Exogenous contaminants, *e.g.*, hitchhiker phosphoproteins from the ABL1-PP and ABL1-Kin^−^ immunopurification, were excluded from the data as the contribution from such proteins only occurred for ‘light’ sites. Consistent with Xue *et al*.^10^, our assay is more robust and sensitive than with normal ATP. The same mass shift (+4 Da) was also observed for the phosphotyrosine-specific immonium fragment ions at *m/z* 216.0420 and *m/z* 220.0505 (Fig. 2b). These ions validate the presence of a tyrosine-phosphorylated peptide. As ion selection is not fully precise, leaching (co-isolation) of contaminating ions with similar *m/z* can occur. Due to the small ∆*m/z*, selection of the ‘heavy’ isotopologue will inadvertently capture the third or fourth isotopic peak of the ‘light’ isotopologue (Fig. 2c). As a consequence, the MS^2^ spectrum will proportionally contain fragment, TMT- and pY-immonium ions originating from both precursor ions. Thus, phosphopeptide assignment and TMT-based quantification can be compromised. To minimise the co-isolation of the ‘heavy’/’light’ phosphopeptide isotopologues and other contaminants, a very narrow precursor ion isolation width of ±0.4 Thomson was chosen. This compromise was selected despite the fact that such a narrow isolation width is considered sub-optimal for the early Q-Exactive generation without the segmented quadrupole^18^,^19^. This setting can lead to a lower identification rate but was considered necessary in this proof-of-principle experiment where the emphasis was on the identification of putative substrates using TMT quantitation. Future applications of HAKA-MS would utilise a larger mass shift of the transferred phosphate group by 6 Da (^18^O_4_-ATP or ^18^O_3_-ATP) combined with newer generation instrumentation that enables precise isolation of the isotopologues without compromising sensitivity. As a quality control measure, the distribution of the pY-immonium ion in individual spectra is ideally suited as an inclusion or exclusion criterion. As ‘light’ pY levels largely remain temporally unaltered, ion leaching can off-set the quantitation of ‘heavy’ pY (Supplementary Fig. 3).

**Figure 3:**
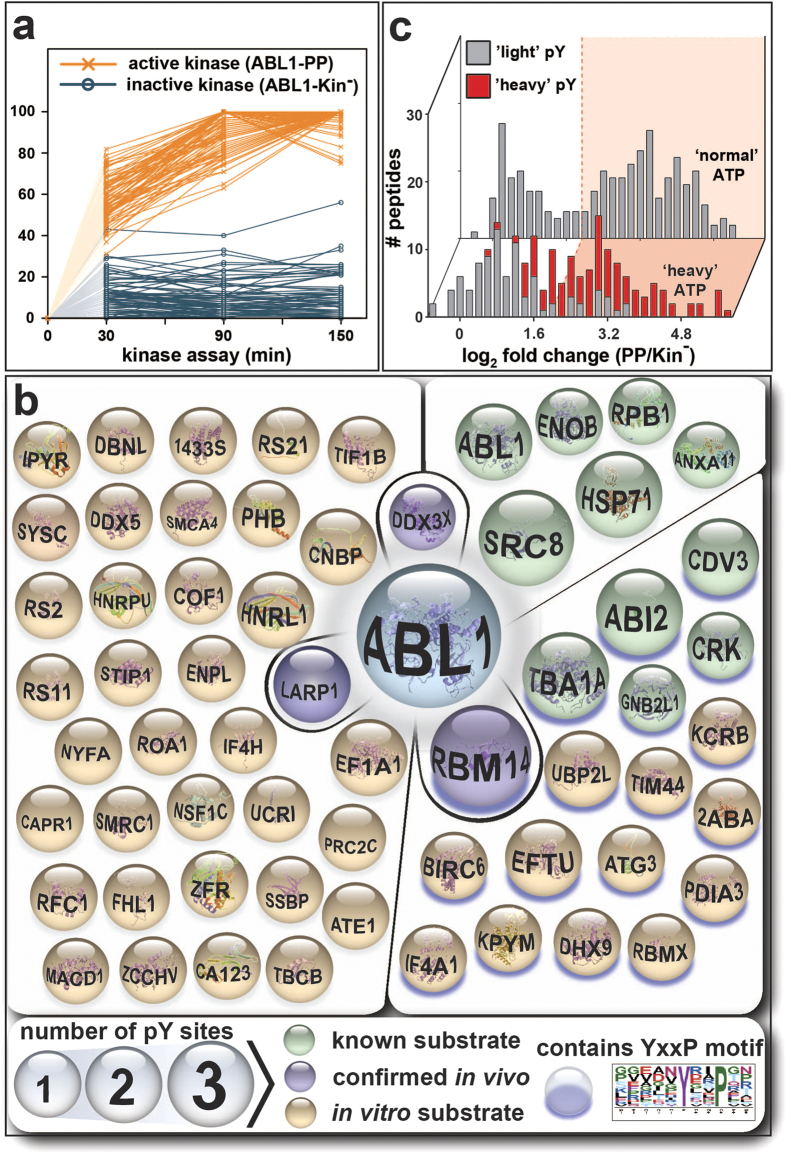
*In vitro* substrates identified by HAKA-MS. (**a**) TMT reporter ion intensities of phosphotyrosine-containing peptides were normalised and plotted against the respective kinase assay time point. Connecting lines are shown for active ABL1-PP (orange) or inactive ABL1-Kin^−^ (blue). As ‘heavy’ phosphate groups are absent prior to the addition of ^18^O-labelled ATP, the lines were extrapolated through the origin (shaded to indicate hypothetical). A clear separation between the kinetic trend lines for active and inactive ABL1 was apparent. (**b**) All 61 identified *in vitro* substrates of ABL1 with at least one ‘heavy’ phosphotyrosine residue. Known and validated substrates are shown in green and purple, respectively. Proteins with purple shading contain the preferred ABL1 YxxP consensus motif (p-value ≤0.05; motif-score: 4.63; fold-increase: 3.16; www.motif-x.med.harvard.edu). Sphere size indicates the number of phosphotyrosines per substrate. (**c**) Comparison of the ABL1-PP/ABL1-Kin^−^ ratio distribution at 90 minutes for HAKA-MS (front) versus the kinase assay performed with normal ATP (back). ‘Light’ and ‘heavy’ pY-containing peptides are labelled in grey and red, respectively. In both cases, the global distribution is bimodal with a shallow, intersecting valley. For the normal ATP kinase assay, the threshold for putative *in vitro* ABL1 substrates was approximated with mixed Gaussian clustering. The first and second clusters are considered noise and *in vitro* substrates, respectively. Comparison to HAKA-MS revealed that regardless of the cut-off stringency (*i.e*., pink, ≥ 4 ratio), data from the normal ATP assay can contain many false positives (grey). Conversely, true positives (red) were excluded.

**Figure 4:**
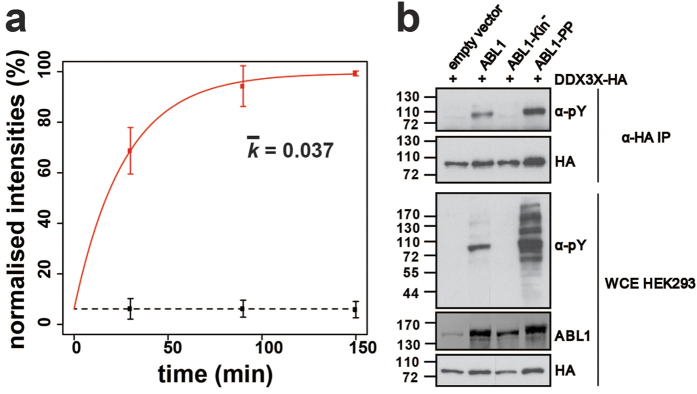
DDX3X is a novel substrate of ABL1. (**a**) Phosphorylation kinetics for the DDX3X peptide DKDAYSSFGSR. The ‘heavy’ phosphate group is located on Y69. Calculated rate constants (*k*) are plotted for the constitutively-active ABL1-PP and catalytically-inactive ABL1-Kin^−^. Error bars represent two standard deviations (2σ) from the mean. (**b**) Biochemical validation of DDX3X tyrosine phosphorylation mediated by ABL1. HA-tagged DDX3X was transiently co-transfected in HEK293 cells in the presence of ABL1, ABL1-Kin^−^, ABL1-PP or the empty vector. DDX3X was immunopurified with HA-beads and the tyrosine phosphorylation state assessed by α-phosphotyrosine (4G10) immunostaining. Total levels of DDX3X-HA, ABL1 and tyrosine-phosphorylated proteins were visualised by immunostaining against α-HA, α-ABL1 and α-phosphotyrosine (4G10), respectively.

**Figure 5:**
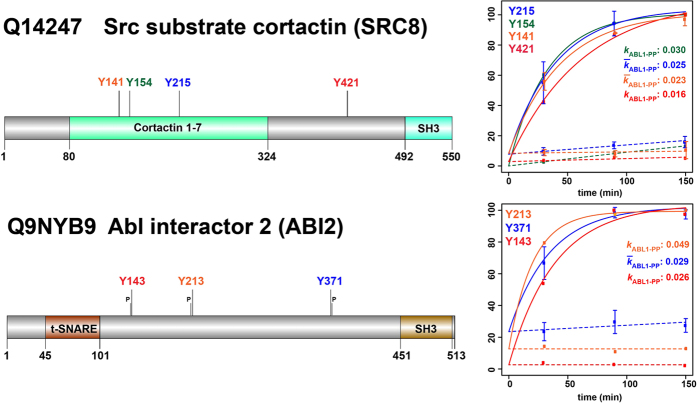
Kinetic rate determination for the multiply-phosphorylated ABL1 substrates SRC8 and ABI2. For Src substrate cortactin (SRC8) and ABL interactor 2 (ABI2), multiple ‘heavy’ pY-sites were identified; with the latter containing the YxxP ABL1 consensus motif at all three sites. The plots show TMT 6-plex-based reaction progress kinetics with different rate constants determined for each of the pY-sites. 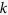 and 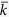, represent rate constant and mean rate constant, respectively. Error bars represent two standard deviations (2σ) from the mean.

The TMT 6-plex data were used to: (i) form individual ABL1-PP/ABL1-Kin^−^ ratio pairs (Supplementary Fig. 4); and (ii) plot normalised intensity-based scatter plots (Fig. 3a, Supplementary Fig. 5). Whilst the ratios showed fold-differences between active versus inactive ABL1 for individual time points, the latter approach enabled the determination of kinetic rates. As expected, ‘light’ and ‘heavy’ pY-sites showed distinct global temporal patterns. The majority of the ‘light’ sites remained unaltered except for a general 1.9-fold offset for active ABL1 (Supplementary Fig. 4). This asymmetry probably arises from interferences introduced by kinase purification and/or the effect of isotopologue pY-ion co-isolation. In contrast, the ‘heavy’ pY-sites showed a clear divergence between active and inactive ABL1 phosphorylation (Fig. 3a). Whilst the inactive controls remained relatively stable, the levels rapidly increased for active ABL1-PP. Not surprisingly, pY-phosphorylation followed an asymptotic trend characteristic of reaction progress kinetics^20^. Due to significant differences, these newly-phosphorylated proteins represent *in vitro* substrates of ABL1. (Fig. 3b, Supplementary Table S1). Of these, 18% are known ABL1 substrates (11 from 61 proteins) and the remaining 82% (50 proteins) constitute potential new substrates identified by HAKA-MS. DDX3X, LARP1 and RBM14 were confirmed as new ABL1 substrates in mammalian cells (Fig. 4, Supplementary Fig. 6). A larger fraction of 18 proteins (30% of the total) were phosphorylated at the preferred ABL1 YxxP consensus motif^21^, a criterion that is often used to further increase confidence in identified substrates from *in vitro* kinase assays. For 13 proteins, multiple *in vitro* pY-sites were identified as illustrated for SRC8 and ABI2 (Fig. 5). It is important to note that *in vitro* kinase assays performed with cell extracts have an inherent risk of observing unusual protein-substrate interactions. This is because non-endogenous concentrations of the active kinase are usually used and the cellular structure is removed. Therefore, identified substrates are generally termed ‘*in vitro*’. As extensively discussed by Knight *et al*.^3^, it is advisable to additionally confirm *in vivo* biological relevance by biochemical and/or other proteomic approaches.

Analogous to Molden *et al*.^11^, HAKA-MS enabled determination of site-specific phosphorylation rates; thus providing potential insights into the *modus operandi* of the kinase. Additional time points using TMT 10-plex would improve the precision of kinetic rate determination. To ascertain if conversion rates were dependent on protein abundance in the cytosolic extract, rate constants were plotted against the relative abundance using the label-free top3 methodology^22^. No correlation was apparent, therefore substrate phosphorylation is not biased by elevated levels of kinase and/or substrates (Supplementary Fig. 7).

### Monitoring Phosphorylation Dynamics via TMT-multiplexing

Published MS-based *in vitro* kinase assays usually utilise normal ATP and single time point measurements which are vulnerable to biochemical and technical variances as illustrated in Fig. 3c for the 90 minute time point. When the assay was performed with ‘light’ ATP only (Supplementary Table S2), the observed bimodal distribution was remarkably similar to HAKA-MS. In order to establish a threshold for putative substrates, a Gaussian cluster analysis was applied. Two interdigitating Gaussian clusters were observed with maxima at ratios of 1.34 and 9.45. These are considered as noise and potential substrates, respectively. An arbitrary cut-off of ≥4 was chosen for the list of putative substrates. Irrespective of such a stringent threshold, the HAKA-MS histogram revealed that when only normal ATP is used, the data can still contain many false positives (grey). As also previously reported^10^,^13^, *in vitro* kinase assay-derived substrate numbers for a single kinase are overestimated. Conversely, the sensitivity of the assay is reduced as potential *in vitro* substrates with lower conversion rates (fold increases) are removed (red).

## Discussion

To avoid *in vitro*-related artefacts, it is vital to maintain protein structure and post-translational modifications. FSBA kinase inactivation minimally affects the native state of the proteins; whereas phosphatase and heat treatment^10^ should be avoided as ABL1 recognition of substrates is partially coordinated via the phosphotyrosine-binding SH2-domain^23^,^24^. One potential drawback of preserving the phosphorylation state, however, is a reduced sensitivity towards substrates that are already present in the cells in the major phosphorylated form. As a consequence, the stoichiometry of the ‘light’ and ‘heavy’ isotopologues and the overall abundance of the phosphopeptides will determine if the peptides can be detected by LCMS under data-dependent conditions. In an attempt to overcome this drawback, a moderate de-phosphorylation of a phosphoprotein-enriched cell fraction prior to the *in vitro* kinase assay has been suggested^10^. Here, an increased sensitivity for ERK1 substrate identification by siKALIP was reported. Such harsh lysate treatments may be required for highly-active serine/threonine kinases, however, the necessity of de-phosphorylated cellular extracts has a minor impact for tyrosine kinases considering that cells show very low levels of tyrosine phosphorylation^24^,^25^. In agreement, Xue *et al*. and others have shown that this approach did not improve phosphotyrosine peptide detection and therefore should not be a limiting factor^10^,^25^,^26^.

Additionally, purification of ABL1-PP and ABL1-Kin^−^ directly from cells maintains the physiological cellular environment of the kinases; *i.e.*, interactions with essential protein cofactors. Compared to existing approaches^3^,^10^, HAKA-MS increases the reliability and confidence of *in vitro* substrate identification by dissecting new phosphorylation sites from the basal phosphoproteome, and integrating phosphorylation rate kinetics for the inactive and active kinase. The latter provides an additional qualitative criterion to discriminate direct ABL1 kinase substrates from potential secondary effects caused by non-inhibited kinase activity or non-kinase-related effects. Additionally, the observed phosphorylation dynamics may provide valuable insights into the sequence of intramolecular phosphorylation events and could be theoretically extended beyond solely comparing active versus inactive kinase. Pre-existing phosphorylation sites can act as modular binding domains, determining subsequent substrate-kinase interactions^27^. Therefore, the preservation of the phosphorylation state in conjunction with native kinase complexes is advisable which is in stark contrast to the siKALIP method^10^. Thus, HAKA-MS is highly beneficial as readily-available technologies are merged and many pitfalls of MS-based *in* vitro kinase assays are circumvented. Ultimately, it is envisaged that the approach will be generically applicable to all kinases to refine and re-define the kinase-substrate landscape.

## Methods

### DNA constructs, antibodies and reagents

ABL1, ABL1-Kin^−^ and ABL1-PP were cloned into pSGT plasmids as previously described^28^. HA-DDX3X was cloned into a pIE plasmid using the Gateway cloning strategy (Invitrogen, Carlsbad, CA, USA). ABL1 antibody (24–21) was produced in-house. The antibody to detect phosphotyrosine proteins (4G10) was initially produced in-house and lately purchased from Millipore (05-777). α-LARP1 (ab86359), α-RBM14 (ab70636), α-tubulin (DM1A), and α-HA-7 (ab49969) were purchased from Abcam (Cambridge, UK), Santa Cruz (Dallas, TX, USA), and Sigma-Aldrich (St. Louis, MO, USA), respectively.

### Cell culture and transfection

HEK293 cells were cultivated in DMEM (GE Healthcare, Little Chalfont, UK), supplemented with 10% foetal calf serum (FCS) (LifeTechologies, Carlsbad, CA, USA), antibiotics (penicillin, 100 U/mL and streptomycin, 100 μg/mL; GE Healthcare, Chalfont St Giles, UK). HEK293 cells were transiently-transfected using Polyfect (Qiagen Inc., Valencia, CA, USA) according to the instructions supplied by the manufacturer and harvested 36 h after transfection.

### Immunopurification and immunoblotting

Cells were lysed in IP-buffer (50 mM Tris-HCl, SIGMA-Aldrich, St.Louis, MO, USA and Merck KGaA, Darmstadt, Germany) pH 7.5, 150 mM NaCl (Merck KGaA), 5 mM EDTA (SIGMA-Aldrich), 5 mM EGTA (SIGMA-Aldrich), 1% NP-40 (Merck KGaA) complemented with protease and phosphatase inhibitors (1 mM Na_3_VO_4_ (SIGMA-Aldrich), inhibitor cocktail (Roche Applied Sciences, Indianapolis, IN, USA), 50 mM NaF (SIGMA-Aldrich), 0.1 mM PMSF (phenylmethanesulfonyl fluoride) (SIGMA-Aldrich), 0.005 mg/mL TPCK (tosyl phenylalanyl chloromethyl ketone) (Sanova Pharma GesmbH, Vienna, Austria) to obtain protein whole cell extracts (WCE). For the cytoplasmic extraction, cells were harvested and lysed on ice for 5 min in buffer N (300 mM sucrose, 10 mM HEPES pH 7.9, 10 mM KCl, 0.1 mM EDTA, 0.1 mM EGTA, 1 mM DTT, 0.75 mM spermidine, 0.15 mM spermine, 0.1% nonidet P-40, 50 mM NaF, 1 mM Na_3_VO_4_, protease inhibitors). The resultant supernatant containing the cytoplasmic fraction was separated from the pelleted nuclei and collected in a fresh tube. Protein content was determined using the Bradford assay (Bio-Rad, Hercules, CA, USA) with γ-globin (BioRad) as the standard.

For the anti-HA immunoprecipitation, beads directly conjugated to HA-7 antibody (Sigma-Aldrich) were incubated with the protein extracts for 2 h on a rotating wheel at 4 °C, washed 3× in IP-buffer and the bound material boiled and eluted in Laemmli buffer (4×). For the immunoprecipitation of endogenous LARP1 and RBM14, 2 mg of pre-cleared protein extracts were incubated with 1 μg of the respective antibody and incubated for 2 h on a wheel at 4 °C. Subsequently, 50 μL of potein G sepharose beads (GE Healthcare) were added and the samples incubated for an additional hour at 4 °C. The beads were washed 3× in IP-buffer, resuspended in Laemmli buffer and boiled 5 min at 95 °C to elute the bound material.

Proteins were electrophoresed by SDS-PAGE and transferred to nitrocellulose membranes (GE-Healthcare, Chalfont St Giles, Buckinghamshire, UK). After blocking with 5% milk or 3% BSA, membranes were incubated for 2 h at RT or overnight at 4 °C with the selected antibodies. Protein signals were visualised through secondary antibodies conjugated to HRP enzyme (Jackson Immunoresearch Laboratories, West Grove, PA, USA) and visualised by ECL immunoblotting detection reagent (GE Healthcare). Alternatively, secondary antibodies conjugated to IRdye (Rockland Immunochemicals Inc., Limerick, PA, USA) were used and signals were detected using the Odyssey infrared imaging system (LI-COR, Lincoln, NE, USA).

### Preparation of FSBA-treated cytosolic fractions

Approximately 30 mg of cytosolic fraction was thawed on ice and treated at a concentration of 10 mg/mL with 5′-[p-(fluorosulfonyl)benzoyl]adenosine (FSBA) in DMSO at a final concentration of 1 mM FSBA and 3% DMSO for 1 h at 30 °C with gently shaking at 300 r.p.m. Residual, reactive FSBA reagent and low molecular-weight compounds such as endogenous ATP were removed by ultracentrifugation with 15 mL 10 K MWCO Amicon Ultra-4 centrifugal filter units (Merck KGaA) at 4,000 × *g* at RT. Proteins were washed 4× the initial volume with IP-buffer, resolubilised in 0.5 mL IP-buffer, pooled into a fresh tube, mixed with 2× kinase buffer (80 mM Tris-HCl, pH 7.5, 50 mM MgCl_2_ (SIGMA-Aldrich), 2 mM DTT (SIGMA-Aldrich), 2 mM Na_3_VO_4_, 50 mM NaF) and stored on ice.

### Immunopurification and kinase assay

For the kinase assay, ABL1 proteins were immunoprecipitated at a ratio of 3.5 μL ABL1 antibody per 0.8 mg whole cell extract and 65 μL slurry of G-sepharose beads (GE-Healthcare). For each kinase reaction with 5 mg FSBA-treated and desalted cellular protein fraction, an equivalent of ABL1-Kin^−^ and ABL1-PP immunopurified from 3.2 mg total HEK293 cell lysate was prepared. Beads were washed 2× with IP-buffer and 1× with KA buffer (40 mM Tris-HCl pH 7.5, 10 mM MgCl_2_, 1 mM DTT), pooled and resuspended in KA buffer. The beads were equally split into eppendorf tubes, 5 mg FSBA-treated cellular protein fraction added and the kinase reaction initiated by adding ‘light’ (SIGMA-Aldrich) or ‘heavy’ ^18^O_2_-ATP (Organisch-Chemisches Institut, UZH, Switzerland)^29^ at a final concentration of 1 mM. After incubation for 30, 90 and 150 min at RT (~23 °C) on a rotary wheel, the reaction was quenched by the addition of solid urea to a final concentration of 8 M. Samples were vortexed and stored on ice.

### Tryptic digestion and peptide purification

Denatured protein samples were reduced (10 mM DTT), alkylated (55 mM iodoacetamide, IA), residual IA quenched (5 mM DTT) and samples diluted with triethyl-ammonium bicarbonate buffer (TEAB) pH 8 to a final concentration of 4 M urea. Samples were digested with endoproteinase LysC (1:400) for 12 h at 37 °C followed by tryptic digestion (1:50) for another 24 h at 37 °C after further dilution of the urea to 1.5 M. Digestion was quenched with 30% TFA and any precipitate removed by centrifugation at 14,000 r.p.m. for 10 min at 4 °C. Acidified peptides were purified by solid-phase extraction using Sep-Pak classic C18 cartridge purification (Waters, Milford, MA, USA). Peptides were consecutively eluted with an increasing percentage of acetonitrile in 0.1% TFA up to 50% and pooled eluates were snap frozen in liquid nitrogen. Organic solvent was removed by lyophilisation and the peptides were resolubilised in IP-buffer (100 mM Tris-HCl pH7.4, 0.3% NP-40) at approximately 3.5 mg/mL. Insoluble matter were removed by centrifugation at 14,000 r.p.m. prior to the α-phosphotyrosine IP.

### Phosphotyrosine enrichment, TMT 6-plex labelling and IMAC enrichment

Protein G sepharose fast-flow beads (GE Healthcare) were conjugated with equal amounts (12 μg/60 μL slurry) of the anti-phosphotyrosine antibodies P-Tyr-100 (Cell-Signaling Technologies Inc., Denvers, MA, USA), 4G10 (Millipore, Billerica, MA, USA) and PT66 (SIGMA-Aldrich). Washed beads were equally split into six eppendorf tubes and incubated with the resolubilised peptide samples for at least 12 h on a rotary wheel at 4 °C. Beads were washed 1× with IP-buffer with and without NP40, once with 10 mM Tris-HCl pH 7.4 before eluting the phosphotyrosine peptides with 2 × 70 μL 5% formic acid. Organic solvent was removed in a vacuum concentrator at 45 °C until ∼2–4 μL remained and the peptides were re-buffered to 500 mM TEAB pH 8. TMT 6-plex labelling was performed according to the instructions of the manufacturer with the exception that the peptides were dissolved in half of the recommended aqueous buffer solution. The labelling reaction was quenched after 90 min by adding 8 μL hydroxylamine solution. The labelled samples were pooled and the organic solvent removed in a vacuum concentrator. Acidified peptides were purified using solid-phase extraction tips containing reversed-phase POROS10R2 material (Applied Biosystems, Foster City, CA, USA). Washed peptides were eluted with 80% acetonitrile containing 0.1% TFA directly onto freshly-prepared immobilised metal affinity chromatography (IMAC) material according to Ficarro *et al*.^17^. Washed phosphopeptides were eluted from the IMAC material with 1.4% ammonia solution containing 1.5 mM ethylenediaminetetraacetic acid (EDTA). The solvent was removed in a vacuum concentrator until ∼2–4 μL remained and then resuspended in a total of 9–20 μL 5% formic acid for analysis by LCMS.

### Liquid chromatography mass spectrometry

Phosphotyrosine peptides enriched from the kinase assays using ‘light’ ATP were analysed on a hybrid linear trap quadrupole (LTQ) Orbitrap Velos mass spectrometer and with ‘heavy’ ATP on a Q-Exactive instrument (ThermoFisher Scientific, Waltham, MA) using Xcalibur version 2.1.0.1160 and 3.0.63, respectively. Peptides were separated by a dual column configuration (pre- and analytical) either on an Agilent 1200 HPLC nanoflow system (Agilent Biotechnologies, Palo Alto, CA) or a DIONEX Ultimate 3000 nanoflow system (ThermoFisher Scientific) via a nanoelectrospray ion source using a liquid junction (Proxeon, Odense, Denmark). Solvents for LCMS separation of the digested samples were as follows: solvent A consisted of 0.4% formic acid in water and solvent B consisted of 0.4% formic acid in 70% methanol and 20% isopropanol. From a thermostatted microautosampler, 8 μL of the tryptic peptide mixture were automatically loaded onto a trap column (Zorbax 300SB-C18 5μm, 5 × 0.3 mm, Agilent Biotechnologies, Palo Alto, CA) at a flow rate of 20 μL/min. 0.1% TFA was used for loading and washing the pre-column. After washing, the peptides were eluted by back-flushing onto a 18 cm fused silica analytical column with an inner diameter of 50 μm packed with C18 reversed phase material (ReproSil-Pur 120 C18-AQ, 3 μm, Dr. Maisch GmbH, Ammerbuch-Entringen, Germany). The peptides were eluted from the analytical column with a 27 minute gradient ranging from 3 to 30 percent solvent B, followed by a 25 minute gradient from 30 to 70 percent solvent B and, finally, a 7 minute gradient from 70 to 100 percent solvent B at a constant flow rate of 100 nL/min. For the analysis of the cytosolic proteome, approximately 400 μg of a tryptic digest of the cytosolic fraction was separated into 20 fractions by RP-HPLC at pH10 on an Agilent 1200 HPLC system (Agilent Biotechnologies) prior to the analysis by nano-LCMS. Fractions were acidified and solvent removed in a vacuum concentrator at 45 °C until ∼2–4 μL remained. Samples were reconstituted in 5% formic acid and analysed as duplicates on a DIONEX Ultimate 3000 nanoflow system coupled to a Q-Exactive mass spectrometer (ThermoFisher Scientific).

The analysis was performed in a data-dependent acquisition mode using a top 10 method for both instruments. Instrument settings for the LTQ Orbitrap Velos were as follows: precursor ions were fragmented by a hybrid collision-induced dissociation (CID) followed by higher-energy collisional dissociation (HCD). Maximal ion accumulation times were 50 and 250 ms, with target values of 5,000 and 200,000 ions for the LTQ and the C-trap, respectively. FTMS scans were performed at a resolution of 60,000 and 7,500 for MS^1^ and MS^2^, respectively. For the Q-Exactive, the maximal ion accumulation time was 250 ms, with target values of 3 × 10^6^ ions for MS scans at a resolution of 70,000 and 50,000 ions for MS^2^ scans at a resolution of 35,000. Dynamic exclusion for selected ions was 60 s. A single lock mass correction at *m/z* 445.12003 was employed^30^. The threshold for switching from MS to MSMS for the Orbitrap Velos was 5,000 counts and normalised collision energy (NCE) was 35% and 40% for CID and HCD, respectively. For the Q-Exactive, MS^2^ scans were triggered at a 1% underfill ratio (~2,000 counts) with an NCE of either 25% or 28% for unlabelled or TMT-labelled samples, respectively.

### Data analysis

Acquired raw data files were processed using the Proteome Discoverer 1.4.0.288 platform and utilising Mascot (v2.3.02, MatrixScience, London, UK) and Sequest HT database search engines and Percolator validation software node (V2.04) to remove false positives with a false discovery rate (FDR) of 1% under strict conditions. In addition, the phosphoRS 3.0 node was used to validate peptide phosphorylation and assignment of normal ‘light’ phosphorylated sites. For ‘heavy’ phosphorylated sites or Sequest HT search results this computational tool is not available. Searches were performed with full tryptic digestion against the human SwissProt database v2014.03_20140331 (40,055 sequences including isoforms obtained from varsplic.pl^31^ and appended known contaminants) with up to two miscleavage sites. Oxidation (+15.9949 Da) of methionine and phosphorylation (+79.9963 Da) of serine, threonine and tyrosine were set as variable modifications, whilst carbamidomethylation (+57.0214 Da) of cysteine residues and TMT 6-plex labelling of peptide N-termini and lysine residues were set as fixed modifications. For data sets containing ‘heavy’ pY-sites, a modification of ^18^O_2_-phosphorylation (+83.9880 Da) was chosen as a variable modification on tyrosine residues in addition to normal phosphorylation. Orbitrap Velos data were searched with mass tolerances of ±10 ppm and 0.6/0.1 Da on the precursor and fragment ions (CID/HCD), respectively. Q-Exactive data were searched with mass tolerances of ±20 ppm and 0.045 Da on the precursor and fragment ions (HCD only), respectively. Results were filtered to include peptide spectrum matches (PSMs) with Mascot ion scores of ≥17, Sequest HT cross-correlation factor (Xcorr) scores of ≥1.7 and medium and high peptide confidence. Lower peptide thresholds and medium peptide confidence settings were chosen to apply less stringent settings for the candidate list before manual curation. For the ‘light’ phosphorylations on serine, threonine and tyrosine residues, phosphoRS 3.0 was used to compute probability values and all peptides with scores >70 were included except ambiguous phosphorylation site assignments. For all ‘heavy’ pY-peptides, MS^2^ spectra were manually inspected and excluded from the final list if: (i) any ambiguity persisted; (ii) no phosphotyrosine was present; (iii) precursor ion isolation was compromised by contaminating co-eluting isobaric peptide ions; (iv) no phosphotyrosine containing *b*- or *y*-fragment ions were detected; (v) no phosphotyrosine-specific immonium ions were detected; or (vi) no ‘heavy’ phosphotyrosine-specific immonium ion was detected. TMT-reporter ion intensities were extracted with a centroid mass tolerance of 80 ppm that was determined from the smallest delta mass deviation from theoretical. Raw TMT intensity values were either used to determine peptide ratios; or were normalised to the most intense reporter ion and subsequently used to generate scatter plots and kinetic rate values (see supplementary material). Unless otherwise stated, the mean value and the respective standard deviation was calculated for multiple spectra of identical PSMs. Further data processing was achieved with R Studio (v0.98.1091), GraphPad Prism (v6.01) and Windows Office Excel (v2013).

### Data availability

The mass spectrometry data have been deposited to the ProteomeXchange Consortium with the identifier PXD002133 and can be accessed via the user name, reviewer23097@ebi.ac.uk; and the password, 6ZiwAkNn.

## Additional Information

**How to cite this article**: Müller, A. C. *et al*. Identifying Kinase Substrates via a Heavy ATP Kinase Assay and Quantitative Mass Spectrometry. *Sci. Rep.* **6**, 28107; doi: 10.1038/srep28107 (2016).

## Supplementary Material

Supplementary Information
