# Supplementary material for: Identifying Kinase Substrates via a Heavy ATP Kinase Assay and Quantitative Mass Spectrometry
Source: Sci Rep. 2016 Jun 27;6:28107. doi: 10.1038/srep28107 (PMC4921819; doi:10.1038/srep28107)
Supplement: Supplementary Information [file srep28107-s1.pdf]

SUPPLEMENTARY MATERIAL

Identifying Kinase Substrates via a Heavy ATP Kinase Assay and Quantitative Mass Spectrometry

André C. Müller,[1#] Roberto Giambruno,[1#$] Juliane Weißer,[1] Peter Májek,[1] Alexandre Hofer,[2] Johannes W. Bigenzahn,[1] Giulio Superti-Furga,[1] Henning J. Jessen,[2]* Keiryn L. Bennett [1]*

SUPPLEMENTARY EXPERIMENTAL PROCEDURES

Modelling of phosphorylation kinetics. Uncorrected TMT reporter ion intensities of all manually-verified MS^2 spectra matching a 'heavy' phosphorylated peptide were normalised against the most intense reporter channel of the spectra to obtain relative reporter intensities $I_S(t)$ of a spectrum *S*. The phosphorylation profiles $I_S(t)$'s corresponding to a phosphorylation site *P* are then modelled as a first order kinetic reaction:

$$I_S(t) = A_P(1 - \mathrm{e}^{-k_P t}) + \epsilon_P$$

where A_P is the saturated, relative phosphorylation at site *P* at time infinity, and ϵ_P corresponds to the measured basal 'heavy' phosphorylation of site *P* at time 0. Due to isotopic impurity of TMT reagents, co-isolation of isobaric peptide ions, chemical or electrical noise in general; $\epsilon_P \neq 0$. The two parameters, A_P and ϵ_P, are shared by the two kinases and only the rate constant, k_P, is kinase specific (k_P^+,k_P^-). Thus, for each phosphorylation site *P*, there are four parameters A_P, ϵ_P, k_P^+ and k_P^- that are fit to the relative intensity data of all corresponding spectra by least square method enforcing all parameters to be non-negative.[1] Calculated relative phosphorylation rates of ABL1-PP, k_P^- for all 'heavy' phosphorylated sites (with dimensions of % per minute) are reported in Supplementary Table 1.

Determination of relative abundance of proteins in the cytosolic fraction using the MS^1-based Top3 methodology. Relative protein abundances in the cytosolic fraction were approximated by using the MS^1-based label-free Top3 peptide approach.[2] Average MS^1 peak areas for the top 3 most intense peptide peaks were reported for each protein (group) using the respective quantitation node in Proteome Discoverer 1.4. Obtained values were used without normalisation against the total sum of all 7,310 identified protein groups.

SUPPLEMENTARY REFERENCES

1 Nelder, J. A. & Mead, R. A Simplex Method for Function Minimization. *The Computer Journal* **7**, 6, doi:10.1093/comjnl/7.4.308 (1965/01/01).

2 Silva, J. C., Gorenstein, M. V., Li, G. Z., Vissers, J. P. & Geromanos, S. J. Absolute quantification of proteins by LCMSE: a virtue of parallel MS acquisition. *Mol Cell Proteomics* **5**, 144-156, doi:10.1074/mcp.M500230-MCP200 (2006).

SUPPLEMENTARY TABLE LEGENDS

Supplementary Table S1: Identified phosphotyrosine-containing peptides using $^{18}O_2$-labelled ATP. Summary of identified and manually-inspected peptide sequences plus fundamental LCMS-related information. Using the one letter code, the type of phosphorylation is indicated in the column 'phosphorylation modification'; and the respective position is given in the 'peptide-sequence'. If more than one peptide-to-spectrum match (PSM) was available, sequence specific information for the highest-scoring spectrum is complemented with the mean and standard deviation of all obtained spectra for TMT 6-plex based quantitation. Abbreviations: Exclusion criterion = peptide excluded after manual inspection; C = strong contamination within precursor isolation window; I(A) = no pY-immonium ion detected; I(B) = no 'heavy' pY-immonium ion detected; F = no pY-spanning (b-/y-)fragment ion detected; YxxP motif = preferred ABL1 consensus motif; *m/z* = mass-to-charge ratio; # scan = instrument scan number of highest scoring spectrum; # PSM = number of peptide-to-spectrum-matches (spectral counts); PP/ Kin^- = TMT reporter ion ratio of active ABL1-PP/ inactive ABL1-Kin^-; STDEV = standard deviation; SP-PP/ Kin^- = single peak detected for ABL1-PP or ABL1-Kin^-; n/a = not applicable.

Supplementary Table S2: Identified phosphotyrosine-containing peptides using normal ATP. Summary of identified peptide sequences plus fundamental LCMS-related information. Using the one letter code, the type and position of phosphorylation is indicated in the column 'phosphorylation modification'. If more than one peptide-to-spectrum match (PSM) was available, sequence specific information for the highest-scoring spectrum is complemented with the mean and coefficient of variation for all obtained spectra for TMT 6-plex based quantitation. Abbreviations: *m/z* = mass-to-charge ratio; # scan = instrument scan number of matched spectrum; # PSM = number of peptide-to-spectrum-matches (spectral counts); PP/ Kin^- = TMT reporter ion ratio of active ABL1-PP/ inactive ABL1-Kin^-; CV = coefficient of variation; n/a = not applicable.

SUPPLEMENTARY FIGURE LEGENDS

Supplementary Figure 1: Kinase inactivation by FSBA. Residual kinase activity was monitored by performing an *in vitro* kinase assay after treatment of a whole cell extract (WCE) with 5'-[p-(fluorosulfonyl)benzoyl] adenosine (FSBA). WCE of HEK293 cells over-expressing the constitutively-active ABL1-PP kinase was incubated with increasing concentrations of FSBA for 1 hour at 37°C. Excess FSBA was removed by ultrafiltration using 10 K MWCO micro-spin filter tubes prior to the kinase assay. Visualisation of phosphotyrosine proteins by SDS-PAGE and α-pY immunoblotting is shown. ABL1 was used as loading control.

Supplementary Figure 2: Immunoblotting of *in vitro* ABL1-PP kinase activity. Time-course experiment monitoring the increase in cellular tyrosine phosphorylation mediated by ABL1-PP kinase activity. Equal volumes of immunoprecipitated, constitutively-active ABL1-PP-beads were incubated with WCE in the presence of 1 mM ATP. The kinase reaction was quenched at the respective time points with gel loading buffer and proteins were separated by SDS-PAGE. Phosphotyrosine-containing proteins were visualised by α-pY immunoblotting. Tubulin was used as loading control.

Supplementary Figure 3: Impact of contaminant ion 'leaching' (co-isolation) on TMT 6-plex-based quantitation. Schematic drawing illustrating the impact that partial co-isolation of 'light' phosphotyrosine-containing ions has on TMT intensity-based quantitation of a 'heavy' phosphotyrosine-containing peptide. For simplification, the following scenario was assumed: (i) for 'heavy' pY-sites there is a strong phosphorylation progression kinetic with no activity detected for the control; and (ii) for 'light' pY-sites, the pre-existing pool of 'light' phosphorylated peptides remains temporally unaffected by HAKA-MS. As an example, if the isolation of a precursor ion results in an 80:20 mixture of 'heavy' and 'light' pY-peptide ions, these will proportionally contribute to all peptide sequence fragment ions, TMT reporter ions and pY-immonium ions. Ultimately, this 'leaching' into the 'heavy' peptide spectrum will offset

the baseline and increase technical noise. The curve-fitting algorithm, however, can account for such baseline shifts and accordingly adapts the position of the origin.

Supplementary Figure 4: TMT ratio distribution of detected phosphotyrosine peptides. Alternative representation of the data shown in **Figure 3a** for 'light' and 'heavy' phosphotyrosine-containing peptides. Ratios for the active versus inactive ABL1 reactions at 30, 90 and 150 minutes were transformed to a $\log_2$ scale and plotted as a frequency distribution. In order to restrict the range of the x-axis, all ratios with a fold change >16 were included in the last bin.

Supplementary Figure 5: Temporal changes in 'light' phosphotyrosine sites identified by HAKA-MS. TMT reporter ion intensities of 'light' phosphotyrosine peptides were normalised and plotted against the respective kinase assay time point (**Figure 3a**). Connecting lines are shown either for active ABL1-PP (orange) or inactive ABl1-Kin$^-$ (blue). As no experimental quantitative data is available for time point zero trend lines are not extrapolated.

Supplementary Figure 6: LARP1 and RBM14 are confirmed as novel ABL1 substrates. Endogenous LARP1 and RBM14 were independently immunopurified from HEK293 cells expressing either ABL1-Kin$^-$ or ABL1-PP. The tyrosine phosphorylation levels of the two proteins were assessed by α-phosphotyrosine (4G10) immunostaining. The respective total levels were subsequently visualised by immunostaining with α-LARP1 or α-RBM14.

Supplementary Figure 7: Correlation of rate constants with the relative abundance of the respective substrate proteins. Relative protein abundances in the cytosolic fraction were approximated by using the Top3 approach. Top3 area values obtained for the cytosolic fraction were plotted against the calculated delta kinetic rate ($k_{ABL1\text{-}PP} - k_{ABL1\text{-}Kin^-}$) for the identified *in vitro* ABL1 kinase substrate peptide sites. Excluding peptides that were only identified from a single tandem mass spectrum, data is averaged values. ABL1 substrates SRC8 and ABI2 are highlighted in red.

SUPPLEMENTARY FIGURES

Supplementary Figure 1

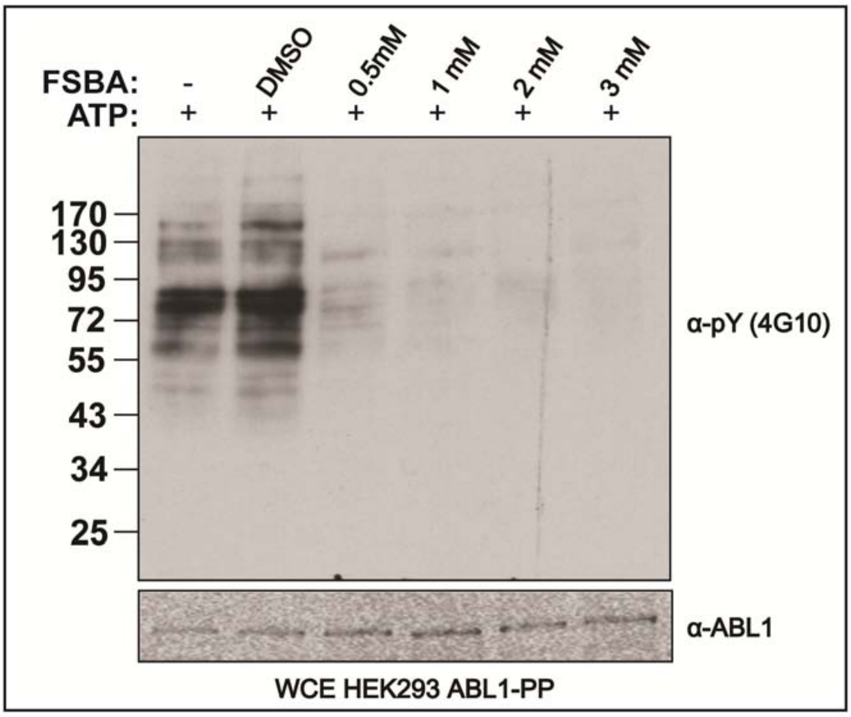

Supplementary Figure 2

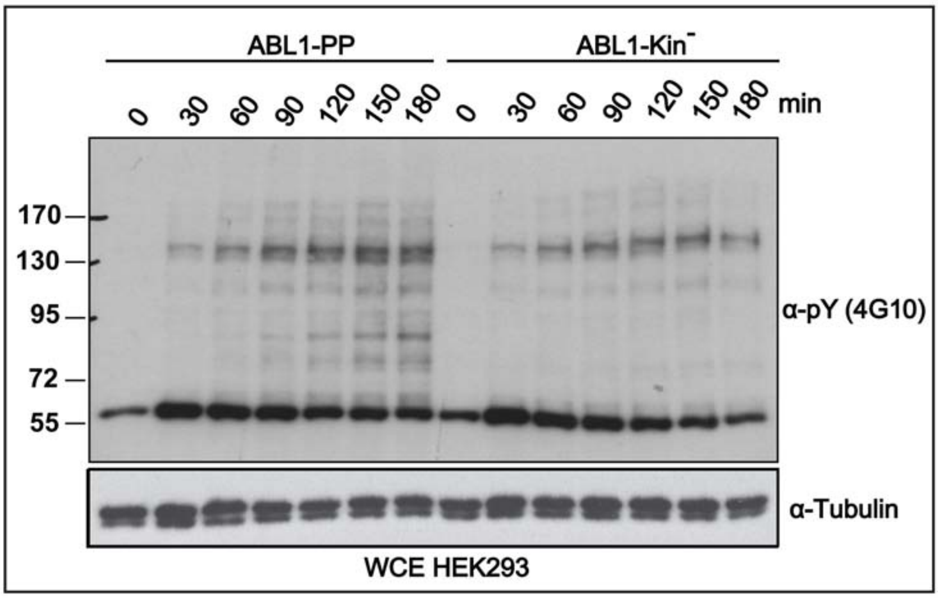

Supplementary Figure 3

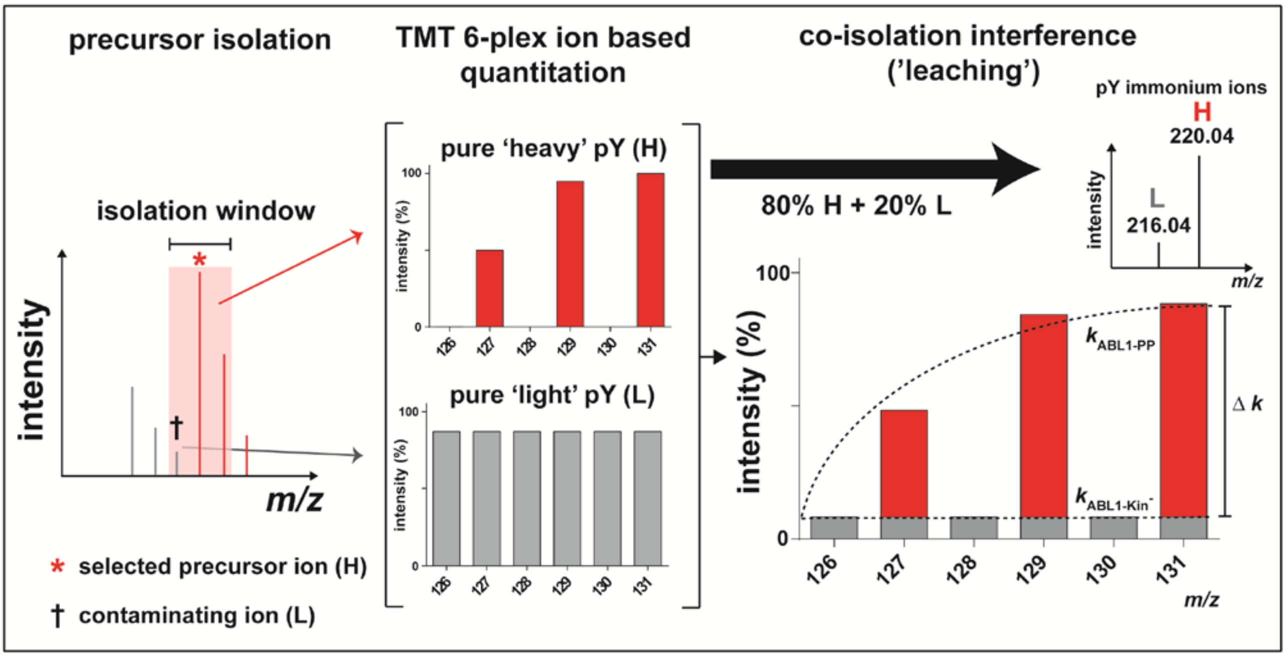

Supplementary Figure 4

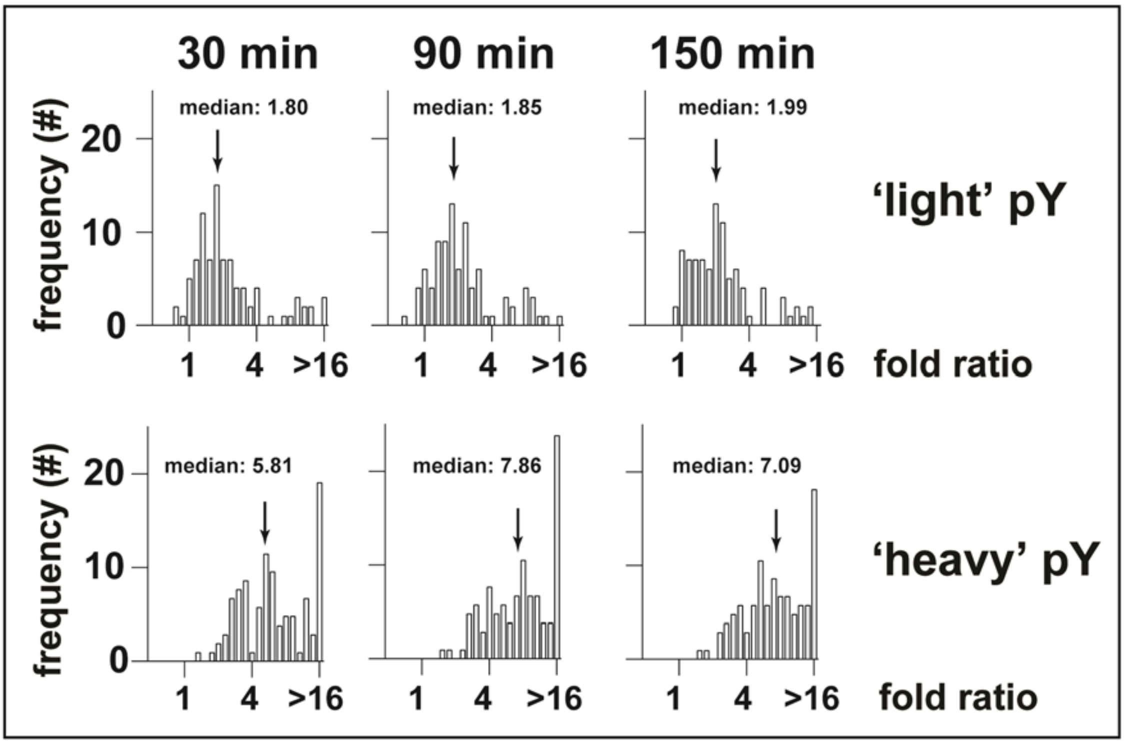

Supplementary Figure 5

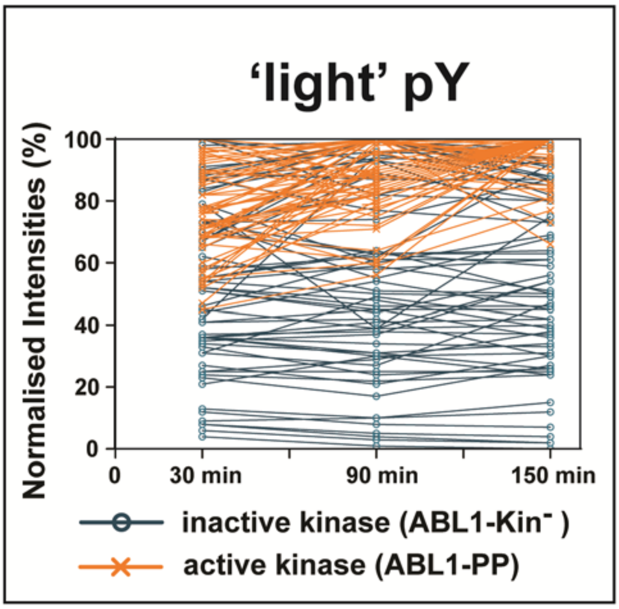

Supplementary Figure 6

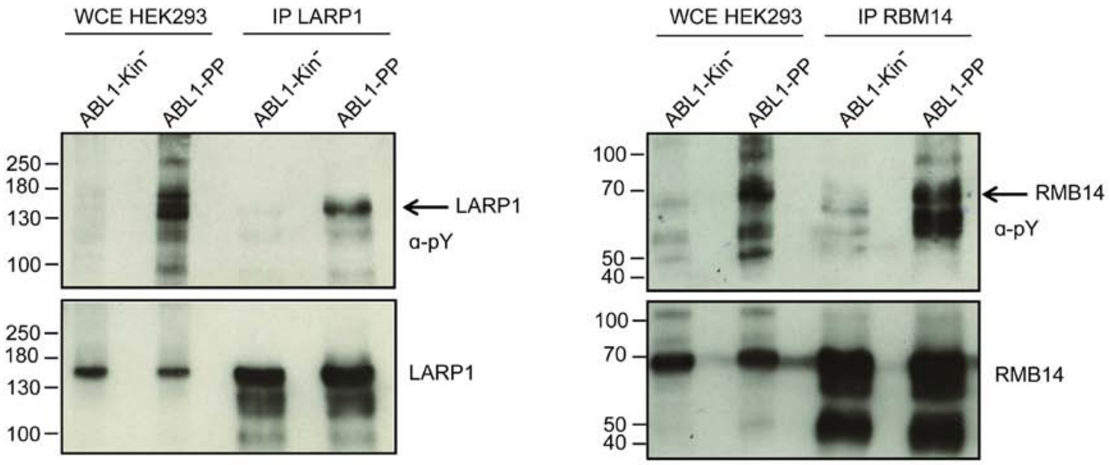

Supplementary Figure 7

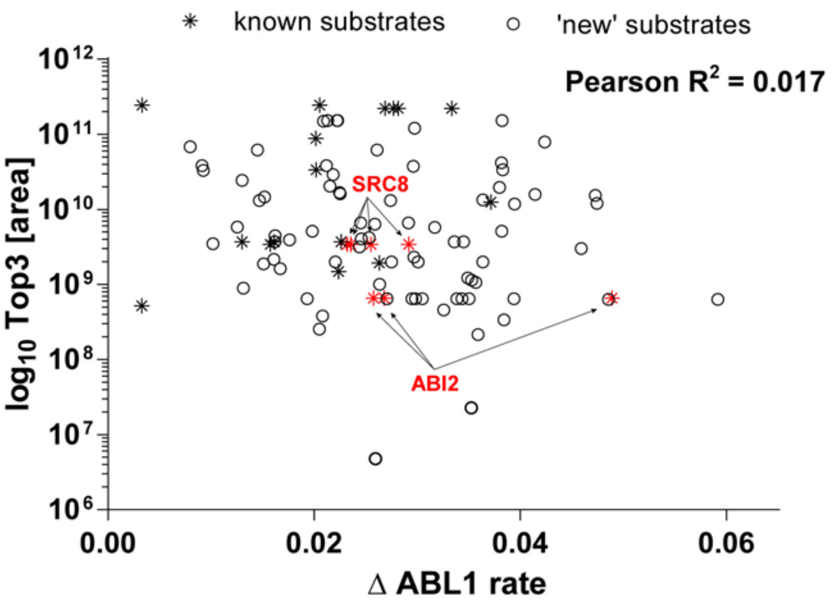

'Heavy' phosphotyrosine peptides

peptide - sequence	Exclusion criterium	YxxP motif	phospho site(s)	protein descriptions	gene name	synonyms	ABL1 substrate	swissprot protein group accessions	phosphorylation modification	phosphoRS Score	Mascot Ion Score	Sequest XCorr	peptide charge	m/z [Th]	retention time (min)	# scan	# PSM	30 min 127/126 PP/Kin^-	STDEV	90 min 129/128 PP/Kin^-	STDEV	150 min 131/130 PP/Kin^-	STDEV	active kinase rate	STDEV	inactive kinase rate	STDEV
dkDAySSFGSR		N	Y69	Isoform 2 of ATP-dependent RNA helicase DDX3X	DDX3X	DDX3X		O00571-2	Y5([18O2]P)	n/a	17		3	592.2898	51.65	6881	2	14.97	11.41	18.13	11.04	20.01	10.88	0.0372	0.01202	0.0000	0.00002
kEIDDSVLGQTGPyR		Y (rP)	Y201	Mitochondrial import inner membrane translocase subunit TIM44	TIMM44	TIM44		O43615	Y14([18O2]P)	n/a	26		3	740.7202	70.23	10885	2	3.78	1.11	5.31	1.75	5.50	2.11	0.0278	0.00169	0.0003	0.00016
eEIVDkyDLFVGSQATDFGEALVR	I(A)	N	Y294	Calumenin	CALU	CALU		O43852	Y7([18O2]P)	n/a	22		3	1081.8848	86.22	14379	1	7.69		8.47		10.46		0.0129		0.0003	
dPSEEAAVLQyASLVGQk		N	Y501	Isoform ATE1-1 of Arginyl-tRNA--protein transferase 1	ATE1	ATE1		O95260-1	Y11([18O2]P)	n/a	23		3	816.4295	82.83	13653	1	2.19		3.71		2.46		0.0387		0.0003	
vLGyNHNGEWcEAQTk		N	Y93	Abl1 human spliceform 1a (myristoylated)	ABL1	Abl1	Y	P00519	Y4([18O2]P)	n/a		2.85	4	612.7919	69.51	10730	1	12.89		47.57		13.10		0.0409		0.0002	
lGGGQyGEVyEGVWk		N	Y253&Y257	Abl1 human spliceform 1a (myristoylated)	ABL1	Abl1	Y	P00519	Y6([18O2]P); Y10([18O2]P)	n/a	46		2	1134.5476	80.20	13077	1	SP-PP		9.66		8.74		0.0374		0.0003	
sINPDEAVAyGAAVQAAILMGDk		N	Y317	Isoform 2 of Heat shock 70 kDa protein 1A/1B	HSPA1A	HSP71	Y	P08107-2	Y10([18O2]P)	n/a	56		3	949.4910	88.37	14842	1	3.49		7.24		6.75		0.0033		0.0000	
eLEQVcNPIISGLyQGAGGPGPGGFGAQGPk		N	Y556	Isoform 2 of Heat shock 70 kDa protein 1A/1B	HSPA1A	HSP71	Y	P08107-2	Y14([18O2]P)	n/a	88		4	900.2040	83.43	13782	1	4.61		5.30		5.19		0.0212		0.0006	
tTPSyVAFTDTER		N	Y41	Isoform 2 of Heat shock 70 kDa protein 1A/1B	HSPA1A	HSP71	Y	P08107-2;P11142-2	Y5([18O2]P)	n/a	43		2	900.9230	66.56	10094	5	6.04	1.00	5.88	1.40	6.69	1.21	0.0217	0.00523	0.0003	0.00008
ekyIDQEELNk	I(B)	N	Y276	Heat shock protein HSP 90-beta	HSP90AB1	HS90A	Y	P08238;P07900-1	Y3([18O2]P)	n/a	19		3	727.3916	64.93	9744	2	3.33	0.40	3.63	0.93	3.13	0.02	0.0225	0.00841	0.0016	0.00005
sSGPYGGGGQyFAkPR		N	Y242	Isoform 2 of Heterogeneous nuclear ribonucleoprotein A1	HNRNPA1	ROA1		P09651-3	Y11([18O2]P)	n/a		2.17	3	724.3662	60.70	8827	1	69.32		39.11		27.64		0.0477		0.0002	
sINPDEAVAyGAAVQAAILSGDk	F	N	Y371	Isoform 2 of Heat shock cognate 71 kDa protein	HSPA8	HSP7C		P11142-2	Y10([18O2]P)	n/a	36		4	701.3682	86.95	14536	1	6.25		10.29		9.20		0.0382		0.0000	
vLTPELyAELR		Y	Y39	Creatine kinase B-type	CKB	KCRB		P12277	Y7([18O2]P)	n/a	25		2	808.9373	82.56	13593	1	6.67		6.86		6.10		0.0304		0.0007	
aAVPSGASTGIyEALELR		N	Y44	Isoform 3 of Beta-enolase	ENO3	ENOB	Y	P13929-3	Y12([18O2]P)	n/a	111		2	1059.5498	84.44	13999	5	2.98	2.39	9.35	4.94	4.88	2.23	0.0206	0.00461	0.0005	0.00039
tATESFASDPILyRPVAVALDTk		Y	Y89	Isoform 3 of Pyruvate kinase isozymes M1/M2	PKM	KPYM		P14618-1/-3	Y13([18O2]P)	n/a	29		4	752.6563	84.65	14044	1	11.35		13.09		27.41		0.0081		0.0001	
dISTNyYASQk		N	Y677	Endoplasmin	HSP90B1	ENPL	Y	P14625	Y6([18O2]P)	n/a		2.3	3	611.3063	61.31	8956	1	2.20		2.75		2.96		0.0424		0.0000	
sPyQEFTDHLVk		N	Y266	40S ribosomal protein S2	RPS2	RS2		P15880	Y3([18O2]P)	n/a	19		3	669.3442	77.97	12587	2	13.12	8.33	23.06	0.08	22.27	1.61	0.0383	0.00755	0.0000	0.00002
gySSLLk		N	Y518	Probable ATP-dependent RNA helicase DDX5	DDX5	DDX5		P17844	Y2([18O2]P)	n/a		1.94	2	655.3691	70.13	10863	1	16.04		22.37		17.61		0.0396		0.0001	
aENyDIPSADR	I(A)	Y	Y873	Ubiquitin-like modifier-activating enzyme 1	UBA1	UBA1		P22314	Y4([18O2]P)	n/a	24		2	782.3553	53.56	7289	1	2.48		6.04		3.67		0.0100		0.0008	
iPLPGAEMLEEEPLyVNAk		N	Y266	Isoform Short of Nuclear transcription factor Y subunit alpha	NFYA	NFYA		P23511-1	Y15([18O2]P)	n/a	31		3	885.7991	86.03	14340	1	4.38		2.58		3.14		0.0279		0.0019	
eILVGDVGQTVDDPyATFVk		N	Y68	Cofilin-1	CFL1	COF1		P23528	Y15([18O2]P)	n/a	84		3	903.4738	82.76	13637	2	5.14	2.54	8.22	4.54	12.36	10.66	0.0261	0.00742	0.0000	0.00000
hELQANcyEEVkDR	I(B)	N	Y140	Cofilin-1	CFL1	COF1		P23528	Y8([18O2]P)	n/a		2.08	3	778.3757	52.79	7123	1	3.05		4.05		5.23		0.0145		0.0000	
ysPTSPTySPTSPk		y/N	S1924&Y1930	DNA-directed RNA polymerase II subunit RPB1	POLR2A	RPB1	Y	P24928	S2(Phospho); Y8([18O2]P)	n/a		2.65	3	712.3259	52.75	7114	1	2.72		1.55		2.41		0.0039		0.0006	
rLAPEyEAAATR	I(A)	N	Y67	Protein disulfide-isomerase A3	PDIA3	PDIA3		P30101	Y6([18O2]P)	n/a	42		2	830.9235	51.46	6841	2	SP-PP		7.21	1.87	5.85	0.08	0.0093	0.00447	0.0002	0.00029
gFPTIyFSPANk		Y	Y454	Protein disulfide-isomerase A3	PDIA3	PDIA3		P30101	Y6([18O2]P)	n/a	40		3	628.6652	82.74	13633	1	5.32		7.16		7.59		0.0217		0.0005	
nLLSVAyk		N	Y48	Isoform 2 of 14-3-3 protein sigma	SFN	1433S		P31947-2	Y7([18O2]P)	n/a		2.41	2	725.4180	78.98	12810	1	2.70		4.85		3.92		0.0268		0.0001	
aLSVGNIDDALQcySEAlk		N	Y74	Stress-induced-phosphoprotein 1	STIP1	STIP1		P31948	Y14([18O2]P)	n/a	58		3	870.4420	85.69	14266	1	5.62		3.94		4.92		0.0138		0.0008	
lEAAEDIAyQLSR		N	Y249	Prohibitin	PHB	PHB		P35232	Y9([18O2]P)	n/a	40		3	597.9683	86.72	14487	1	5.56		SP-PP		7.17		0.0473		0.0000	
lTPTSVLDyFGTGSVQR		N	Y168	Isoform 2 of Replication factor C subunit 1	RFC1	RFC1		P35251-2	Y9([18O2]P)	n/a	36		3	718.7000	85.84	14299	1	1.32		2.25		1.62		0.0161		0.0029	
dVyLSPR		Y	Y206	Isoform 2 of RNA-binding motif protein, X chromosome	RBMX	RBMX		P38159-1	Y3([18O2]P)	n/a	21		2	581.7963	58.66	8381	1	5.61		8.48		10.57		0.0297		0.0000	
rVPNAyDk		Y	Y251	Isoform Crk-II of Adapter molecule crk	CRK	CRK	Y	P46108-1	Y6([18O2]P)	n/a	28		3	502.2736	47.66	6024	1	11.69		32.23		18.68		0.0226		0.0002	
iAIyELLFk	I(B)	N	Y12	40S ribosomal protein S10	RPS10	RS10		P46783	Y4([18O2]P)	n/a	32		2	826.4845	88.42	14853	1	11.62		16.50		11.03		0.0229		0.0004	
vPDFSEyR		N	Y91	Cytochrome b-c1 complex subunit Rieske, mitochondrial	UQCRFS1	UCRI		P47985	Y7([18O2]P)	n/a		1.99	2	663.3086	70.88	11029	2	18.63	13.37	21.42	3.10	20.16	7.28	0.0210	0.00218	0.0002	0.00007
eAQLyAAQAHLk	C	N	Y540	Nuclear autoantigenic sperm protein	NASP	NASP		P49321	Y5([18O2]P)	n/a	17		3	629.0092	68.04	10413	1	2.71		2.69		3.34		0.0415		0.0000	
gITINAAHVEySTAAR		N	Y115	Elongation factor Tu, mitochondrial	TUFM	EFTU		P49411	Y11([18O2]P)	n/a	32		3	663.0057	67.32	10259	2	6.19	2.69	8.60	0.45	7.03	0.13	0.0278	0.01062	0.0004	0.00025
dLEkPFLLPVEAVySVPGR		Y	Y266	Elongation factor Tu, mitochondrial	TUFM	EFTU		P49411	Y14([18O2]P)	n/a	50		3	891.1619	84.80	14077	5	SP-PP		19.98	23.04	19.57	18.47	0.0149	0.00292	0.0002	0.00016
eVmQEVAQLSQFDEELyk		N	Y248	Serine--tRNA ligase, cytoplasmic	SARS	SYSC		P49591	Y17([18O2]P)	n/a	50		3	915.4501	84.93	14103	1	2.86		2.93		3.63		0.0106		0.0004	
dAQELyAAGENR		N	Y365	Annexin A11	ANXA11	ANXA11	Y	P50995	Y6([18O2]P)	n/a	19		2	825.3798	56.18	7843	1	7.46		3.89		5.37		0.0171		0.0010	
eVDySDSLTEk		N	Y1379	Isoform 5 of Transcription activator BRG1	SMARCA4	SMCA4		P51532-5	Y4([18O2]P)	n/a	36		3	609.9649	62.87	9290	1	7.92		10.67		13.26		0.0353		0.0000	
gIDVQQVSLVINyDLPTNR		Y	Y347	Eukaryotic initiation factor 4A-I	EIF4A1	IF4A1		P60842	Y13([18O2]P)	n/a	48		3	819.7636	86.21	14378	1	7.37		2.90		3.82		0.0402		0.0020	
aDGyEPPVQESV	C	Y	Y256	40S ribosomal protein S3a	RPS3A	RS3A		P61247	Y4([18O2]P)	n/a	31		2	802.3645	60.71	8830	2	1.99	0.08	1.66	0.26	1.79	0.11	0.0255	0.00099	0.0040	0.00080
eAIEGTyIDk		N	Y55	40S ribosomal protein S11	RPS11	RS11		P62280	Y7([18O2]P)	n/a	30		3	560.9603	68.37	10483	2	18.50	15.17	11.88	3.15	9.62	6.83	0.0371	0.00249	0.0007	0.00046
gFQFVSSSLPDIcyR		N	Y48	Isoform 2 of Cellular nucleic acid-binding protein	CNBP	CNBP		P62633-2	Y14([18O2]P)	n/a	34		3	696.9997	84.01	13907	1	3.50		5.14		5.62		0.0198		0.0000	
dcDLQEDAcyNcGR		N	Y75	Isoform 2 of Cellular nucleic acid-binding protein	CNBP	CNBP		P62633-2	Y10([18O2]P)	n/a	35		3	696.9319	52.19	6996	1	2.86		3.80		3.78		0.0382		0.0000	
eEDGRyRDPTTVTTLR		Y	Y143	Isoform 1 of Serine/threonine-protein phosphatase 2A	PPP2R2A	2ABA		P63151-1	Y6([18O2]P)	n/a		2.64	3	741.3619	53.65	7308	2	SP-PP		21.24	4.43	22.99	10.01	0.0162	0.00286	0.0000	0.00004
tyAIcGAIR		N	Y53	40S ribosomal protein S21	RPS21	RS21		P63220	Y2([18O2]P)	n/a	24		2	669.3350	65.07	9774	2	4.80	1.78	7.45	2.89	8.29	3.88	0.0227	0.00445	0.0001	0.00002
lTRDETNyGIPQR		Y	Y52	Guanine nucleotide-binding protein subunit beta-2-like	GNB2L1	GNB2L1	Y	P63244	Y8([18O2]P)	n/a	20		3	625.9816	57.98	8235	1	4.94		6.59		5.86		0.0206		0.0004	
lPLQDVyk		N	Y254	Elongation factor 1-alpha 1	EEF1A1	EF1A1		P68104	Y7([18O2]P)	n/a	36		3	506.6224	77.92	12576	4	36.51	31.39	47.37	21.00	54.43	24.39	0.0224	0.00293	0.0001	0.00006
sTTTGHLIyk		N	Y29	Elongation factor 1-alpha 1	EEF1A1	EF1A1		P68104	Y9([18O2]P)	n/a	23		3	554.9721	58.91	8435	4	9.69	2.12	14.60	5.09	14.95	10.92	0.0226	0.00493	0.0004	0.00030
gyFEYIEENk		N	Y257	Isoform Short of Heterogeneous nuclear ribonucleoprotein U	HNRNPU	HNRPU		Q00839-2	Y2([18O2]P)	n/a	39		3	611.9674	79.88	13006	1	12.69		17.27		15.18		0.0297		0.0001	
sGDSEVyQLGDVSQk		N	Y73	Single-stranded DNA-binding protein, mitochondrial	SSBP1	SSBP		Q04837	Y7([18O2]P)	n/a	57		3	718.6887	73.33	11565	3	3.72	2.19	4.69	2.75	6.24	2.20	0.0247	0.00586	0.0001	0.00015
aENNSEVGASGyGVPGPTWDR		Y	Y132	Isoform 1 of ATP-dependent RNA helicase A	DHX9	DHX9		Q08211-1	Y12([18O2]P)	n/a	71		3	826.0415	69.43	10713	3	3.20	0.84	4.15	0.57	4.56	0.60	0.0259	0.00020	0.0000	0.00035
vFPGSTTEDyNLIVIER		N	Y435	Isoform 2 of Transcription intermediary factor 1-beta	TRIM28	TIF1B		Q13263-2	Y10([18O2]P)	n/a	75		3	756.0508	83.44	13785	1	3.12		5.79		3.58		0.0384		0.0004	
aIVAGDQNVEyk		N	Y117	Isoform 3 of Four and a half LIM domains protein 1	FHL1	FHL1		Q13642-3	Y11([18O2]P)	n/a	42		3	616.9937	61.74	9049	1	4.96		7.47		7.77		0.0317		0.0000	
dGSLASNPySGDLTk		N	Y858	Isoform 4 of Ubiquitin-associated protein 2-like	UBAP2L	UBP2L		Q14157-4	Y9([18O2]P)	n/a	35		3	689.6783	64.98	9756	3	10.58	4.60	18.98	5.62	12.52	5.38	0.0292	0.00466	0.0001	0.00011
fPLDyySIPFPTPTTPLTGR		Y	Y834&Y835	Isoform 4 of Ubiquitin-associated protein 2-like	UBAP2L	UBP2L		Q14157-4	Y5([18O2]P); Y6([18O2]P)	n/a	36		3	894.0955	87.79	14718	1	1.78		2.78		2.90		0.0248		0.0003	
lPSSPVyEDAASFk		N	Y421	Isoform 3 of Src substrate cortactin	CTTN	SRC8	Y	Q14247	Y7([18O2]P)	n/a	39		3	685.0195	79.52	12930	1	11.81		15.10		19.41		0.0159		0.0002	
lPSsPVyEDAASFk		N	S418&Y421	Isoform 3 of Src substrate cortactin	CTTN	SRC8	Y	Q14247	S4(Phospho); Y7([18O2]P)	n/a	34		3	711.6746	80.30	13098	1	7.92		11.65		12.94		0.0237		0.0001	
vDQSAVGFEyQGk		N	Y141	Isoform 3 of Src substrate cortactin	CTTN	SRC8	Y	Q14247	Y10([18O2]P)	n/a	37		3	657.3311	68.46	10502	2	6.43	0.83	11.29	2.90	11.21	6.07	0.0234	0.00421	0.0002	0.00023
dySSGFGGk		N	Y154	Isoform 3 of Src substrate cortactin	CTTN	SRC8	Y	Q14247	Y2([18O2]P)	n/a	21		2	730.3556	56.50	7913	1	23.43		10.89		7.69		0.0301		0.0009	
sAVGFEyQGk		N	Y215	Isoform 3 of Src substrate cortactin	CTTN	SRC8	Y	Q14247	Y7([18O2]P)	n/a	28		2	814.4203	62.48	9206	2	5.72	0.05	7.04	1.75	6.28	1.22	0.0262	0.00669	0.0007	0.00008
dGyQQNFk		N	Y670	Isoform 2 of Caprin-1	CAPRIN1	CAPR1		Q14444-1/-2	Y3([18O2]P)	n/a	33		3	514.5897	52.65	7093	1	16.71		55.60		19.94		0.0327		0.0001	
aySSFGGGR		N	Y12	Isoform Short of Eukaryotic translation initiation factor 4H	EIF4H	IF4H		Q15056-2	Y2([18O2]P)	n/a	18		2	607.7803	48.44	6190	1	5.81		4.95		5.55		0.0159		0.0007	
aAPFSLEyR		N	Y17	Inorganic pyrophosphatase	PPA1	IPYR		Q15181	Y8([18O2]P)	n/a	28		2	683.8406	76.68	12300	2	4.55	0.29	7.60	1.28	7.39	1.55	0.0219	0.00687	0.0001	0.00013
fEPEySQlk		N	Y609	Isoform 2 of La-related protein 1	LARP1	LARP1		Q6PKG0-3	Y5([18O2]P)	n/a	38		3	561.6243	71.81	11233	5	14.09	7.87	11.34	8.35	13.11	7.51	0.0338	0.00939	0.0001	0.00018
sLPTTVPEsPNyR		N	S774&Y777	Isoform 2 of La-related protein 1	LARP1	LARP1		Q6PKG0-3	S9(Phospho); Y12([18O2]P)	n/a	20		3	618.6221	65.04	9768	1	3.33		4.93		4.74		0.0345		0.0000	
sLPTTVPESPNyR		N	Y777	Isoform 2 of La-related protein 1	LARP1	LARP1		Q6PKG0-3	Y12([18O2]P)	n/a	26		3	591.9641	64.45	9636	2	2.78	0.45	4.58	0.03	4.31	0.73	0.0164	0.00032	0.0002	0.00028
eDAANNyAR		N	Y103	Isoform 2 of Tubulin alpha-1A chain	TUBA1A	TBA1A	Y	Q71U36-2	Y7([18O2]P)	n/a	17		2	668.7985	39.31	4262	1	SP-PP		24.34		9.88		0.0283		0.0006	
iHFPLATyAPVISAEk		N	Y272	Isoform 2 of Tubulin alpha-1A chain	TUBA1A	TBA1A	Y	Q71U36-2	Y8([18O2]P)	n/a	59		3	767.0939	82.37	13551	3	4.93	1.67	9.51	2.71	9.38	3.11	0.0269	0.00164	0.0000	0.00011
nLDIERPTyTNLNR	F	N	Y224	Isoform 2 of Tubulin alpha-1A chain	TUBA1A	TBA1A	Y	Q71U36-2	Y9([18O2]P)	n/a		1.79	3	678.0107	68.18	10441	1	8.97		7.82		7.09		0.0289		0.0007	
vGINyQPPTVVPGGDLAk		Y	Y357	Isoform 2 of Tubulin alpha-1A chain	TUBA1A	TBA1A	Y	Q71U36-2	Y5([18O2]P)	n/a	41		3	789.7672	79.29	12878	1	4.67		7.50		5.54		0.0337		0.0003	
gTGLLSSDyR		N	Y410	Isoform 3 of Zinc finger CCCH-type antiviral protein 1	ZC3HAV1	ZCCHV		Q7Z2W4-3	Y9([18O2]P)	n/a	36		2	691.3388	62.81	9279	1	9.58		15.11		13.70		0.0459		0.0000	
kPSyAEIcQR	I(B)	N	Y650	La-related protein 4B BLAST	LARP4B	LARP4B		Q92615	Y4([18O2]P)	n/a	18		3	598.6433	53.04	7178	1	15.95		18.36		14.02		0.0360		0.0003	
nRPPFGQGyTQPGPGyR	C	Y	Y392&Y399	Isoform 2 of Protein TFG	TFG	TFG		Q92734-2	Y9([18O2]P); Y16([18O2]P)	n/a		2.36	3	763.6833	56.75	7968	1	2.83		2.91		2.73		0.0270		0.0016	
kGQASLyGk		N	Y352	SWI/SNF complex subunit SMARCC1	SMARCC1	SMRC1		Q92922	Y7([18O2]P)	n/a		2.29	3	575.0008	52.65	7094	1	5.48		7.97		5.47		0.0364		0.0005	
aQENyEGSEEVSPPQTk	I(A)	N	Y2556	Isoform 2 of Probable ubiquitin carboxyl-terminal hydrolase FAF-X	USP9X	USP9X		Q93008-1	Y5([18O2]P)	n/a	57		3	812.3887	52.97	7161	1	SP-PP		9.47		11.40		0.0245		0.0001	
sTAPAVAyDSk		N	Y155	Zinc finger RNA-binding protein	ZFR	ZFR		Q96KR1	Y8([18O2]P)	n/a	35		2	826.4285	52.50	7061	2	9.03	5.06	7.94	0.88	9.61	4.03	0.0330	0.00322	0.0004	0.00062
lAELSDyR		N	Y614	Isoform 1 of RNA-binding protein 14	RBM14	RBM14		Q96PK6-1	Y7([18O2]P)	n/a	24		2	640.3172	70.98	11050	2	40.84	12.34	49.64	5.44	34.74	3.87	0.0395	0.00047	0.0001	0.00008
rLPDAHSDyAR		N	Y645	Isoform 1 of RNA-binding protein 14	RBM14	RBM14		Q96PK6-1	Y9([18O2]P)	n/a	38		3	538.5975	45.18	5486	4	SP-PP		12.81	8.19	7.49	9.43	0.0346	0.01543	0.0002	0.00045
aQPSASLGVGyR		N	Y261	Isoform 1 of RNA-binding protein 14	RBM14	RBM14		Q96PK6-1	Y11([18O2]P)	n/a	33		2	759.8862	56.66	7948	2	4.99	2.46	5.40	1.44	4.75	1.74	0.0303	0.00365	0.0008	0.00024
aQPSVSLGAAyR	C	N	Y249	Isoform 1 of RNA-binding protein 14	RBM14	RBM14		Q96PK6-1	Y11([18O2]P)	n/a	33		3	511.5988	60.16	8710	2	1.91	0.16	2.73	0.50	2.26	0.26	0.0281	0.00064	0.0010	0.00050
aSyVAPLTAQPATYR		Y	Y226	Isoform 1 of RNA-binding protein 14	RBM14	RBM14		Q96PK6-1	Y3([18O2]P)	n/a	34		3	641.3314	69.46	10719	2	5.18	1.64	4.70	1.11	5.45	2.92	0.0312	0.00365	0.0007	0.00084
aSyVAPLTAQPATyR		Y	Y237	Isoform 1 of RNA-binding protein 14	RBM14	RBM14		Q96PK6-1	Y3([18O2]P); Y14([18O2]P)	n/a	28		3	669.3208	66.62	10107	2	3.26	1.39	3.36	0.11	2.70	0.15	0.0212	0.00196	0.0018	0.00152
lGEyEDVSR		N	Y98	Tubulin-folding cofactor B	TBCB	TBCB		Q99426	Y4([18O2]P)	n/a	29		2	690.8218	56.85	7990	1	5.79		7.86		11.68		0.0176		0.0000	
qREEHyFck		N	Y114	O-acetyl-ADP-ribose deacetylase MACROD1	MACROD1	MACD1		Q9BQ69	Y6([18O2]P)	n/a		1.88	3	613.6318	54.83	7557	1	11.34		13.28		14.54		0.0265		0.0001	

Supplementary Table 1

'Heavy' phosphotyrosine peptides																											
peptide - sequence	Exclusion criterium	YxxP motif	ABL1 substrate	protein descriptions	gene name	synonyms	ABL1 substrate	swissprot protein group accessions	phosphorylation modification	phosphoRS Score	Mascot Ion Score	Sequest XCorr	peptide charge	m/z [Th]	retention time (min)	# scan	# PSM	30 min 127/126 PP/Kin$^-$	STDEV	90 min 129/128 PP/Kin$^-$	STDEV	150 min 131/130 PP/Kin$^-$	STDEV	active kinase rate	STDEV	inactive kinase rate	STDEV
qNQFyDTQVIk		N	Y111	Isoform 3 of Heterogeneous nuclear ribonucleoprotein U-like protein 1	HNRNPUL1	HNRL1		Q9BUJ2-3	Y5([18O2]P)	n/a	43		2	963.4996	72.53	11392	6	SP-PP		32.39	54.02	22.50	23.14	0.0365	0.00476	0.0001	0.00026
gyFEHR		N	Y183	Isoform 3 of Heterogeneous nuclear ribonucleoprotein U-like protein 1	HNRNPUL1	HNRL1		Q9BUJ2-3	Y2([18O2]P)	n/a	22		3	374.5085	44.68	5379	1	SP-PP		4.00		9.49		0.0301		0.0000	
nPPGASTyNk		N	Y729	Isoform 3 of Heterogeneous nuclear ribonucleoprotein U-like protein 1	HNRNPUL1	HNRL1		Q9BUJ2-3	Y8([18O2]P)	n/a	20		3	530.9395	45.70	5598	1	3.27		3.06		3.18		0.0236		0.0015	
vVDYSQFQEsDDADEDyGRDSGPPTk	C	N	Y26	Isoform 1 of Nuclear ubiquitous casein and cyclin-dependent kinase substrate 1	NUCKS1	NUCKS		Q9H1E3-1	S10(Phospho); Y17([18O2]P)	n/a	19		4	886.3820	72.69	11426	1	2.51		3.13		2.55		0.0364		0.0011	
kVVDHyENPR	I(B)	Y	Y43	Isoform 1 of Iron-sulfur cluster assembly enzyme ISCU, mitochondrial	ISCU	ISCU		Q9H1K1-1	Y6([18O2]P)	n/a	23		3	600.3184	45.39	5532	1	8.98		17.63		7.00		0.0213		0.0008	
tAEIVyAATTSLR		N	Y4489	Baculoviral IAP repeat-containing protein 6	BIRC6	BIRC6		Q9NR09	Y6([18O2]P)	n/a	45		3	570.3002	84.20	13948	2	5.98	1.08	7.61	0.44	5.20	0.92	0.0490	0.00602	0.0005	0.00006
dSDQFEWVTIEQSGELVyEAPETVAAEPPPIk	I(B)	Y	Y4130	Baculoviral IAP repeat-containing protein 6	BIRC6	BIRC6		Q9NR09	Y18([18O2]P)	n/a	45		4	1030.0111	85.55	14235	1	3.33		5.94		2.27		0.0609		0.0017	
aLEVAEyLTPVLk		Y	Y18	Isoform 2 of Ubiquitin-like-conjugating enzyme ATG3	ATG3	ATG3		Q9NT62-2	Y7([18O2]P)	n/a	39		3	663.3799	86.60	14462	2	SP-PP		46.72	24.50	60.29	72.04	0.0169	0.00256	0.0002	0.00011
aQESVGIyEVTHQFVk		N	Y151	UPF0587 protein C1orf123	C1orf123	CA123		Q9NWV4	Y8([18O2]P)	n/a	60		3	793.0859	82.91	13670	1	6.66		13.44		9.06		0.0153		0.0002	
kPIDyTILDDIGHGVk		Y	Y98	Isoform 3 of Abl interactor 2	ABI2	ABI2	Y	Q9NYB9-3	Y5([18O2]P)	n/a	44		4	639.6120	84.85	14087	1	13.89		35.08		47.45		0.0258		0.0000	
tLEPVRPPVVPNDyVPSPTR		y	Y162	Isoform 3 of Abl interactor 2	ABI2	ABI2	Y	Q9NYB9-3	Y14([18O2]P)	n/a	27		3	849.4502	76.55	12271	1	5.57		8.95		7.81		0.0489		0.0000	
hTPPTIGGSLPyR		Y (rPP)	Y359	Isoform 3 of Abl interactor 2	ABI2	ABI2	Y	Q9NYB9-3	Y12([18O2]P)	n/a	48		3	570.2979	61.11	8914	5	2.76	0.50	3.17	1.26	3.50	0.50	0.0277	0.01140	0.0009	0.00058
fQDVGPQAPVGSVyQk		N	Y162	Isoform 1 of Drebrin-like protein	DBNL	DBNL		Q9UJU6-1	Y14([18O2]P)	n/a	38		3	754.7295	72.54	11394	1	3.44		4.76		6.02		0.0161		0.0000	
lQLDNQyAVLENQk		N	Y244	Isoform 1 of Protein CDV3 homolog	CDV3	CDV3	Y	Q9UKY7-1	Y7([18O2]P)	n/a	57		3	740.0612	78.06	12609	1	4.50		9.44		6.94		0.0229		0.0003	
tPQGPPEIySDTQFPSLQSTAk		Y	Y190	Isoform 1 of Protein CDV3 homolog	CDV3	CDV3	Y	Q9UKY7-1	Y9([18O2]P)	n/a	76		3	978.8275	79.92	13016	1	8.11		10.13		12.38		0.0130		0.0000	
lGAAPEEESAyVAGEk		N	Y167	Isoform 4 of NSFL1 cofactor p47	NSFL1C	NSF1C		Q9UNZ2-6	Y11([18O2]P)	n/a	39		3	721.6979	69.85	10801	1	2.26		4.08		3.50		0.0263		0.0000	
lPSAQTPNGTDyVASGk		N	Y1971	Isoform 2 of Protein PRRC2C	PRRC2C	PRC2C		Q9Y520-2	Y12([18O2]P)	n/a	28		3	750.0507	66.34	10047	1	3.06		4.24		4.96		0.0349		0.0000	

'Light' phosphotyrosine peptides																											
peptide - sequence			phospho site(s)	protein descriptions	gene name	synonyms		swissprot protein group accessions	phosphorylation modification	phosphoRS Score	Mascot Ion Score	Sequest XCorr	peptide charge	m/z [Th]	retention time (min)	# scan	# PSM	30 min 127/126 PP/Kin$^-$	STDEV	90 min 129/128 PP/Kin$^-$	STDEV	150 min 131/130 PP/Kin$^-$	STDEV	active kinase rate	STDEV	inactive kinase rate	STDEV
vIyDFIEk			Y256	Neural Wiskott-Aldrich syndrome protein	WASL	WASL		O00401	Y3(Phospho)	213	33		3	522.2877	84.60	14033	3	1.13	0.06	1.00	0.03	1.07	0.17				
vRPcVVyGGADIGQQIR			Y301	Isoform 2 of ATP-dependent RNA helicase DDX3X	DDX3X	DDX3X		O00571-2	Y7(Phospho)	534	62		3	733.0447	70.49	10941	2	8.77	1.64	8.63	2.86	9.47	2.66				
eLAVQIyEEAR			Y283	Isoform 2 of ATP-dependent RNA helicase DDX3X	DDX3X	DDX3X		O00571-2	Y7(Phospho)	296	26		2	815.4090	79.39	12900		7.69		5.82		5.11					
vEGDNIyVR			Y623	Isoform 1 of Band 4.1-like protein 2	EPB41L2	E41L2		O43491-1	Y7(Phospho)	305	42		2	687.3366	62.21	9147		1.46		1.52		1.46					
sESVVyADIR			Y263	Isoform 5 of Myelin protein zero-like protein 1	MPZL1	MPZL1		O95297-5	Y6(Phospho)	300	45		2	724.3549	66.20	10016		1.82		1.84		1.70					
sDDymPmSPASVSAPk			Y675	Insulin receptor substrate 2	IRS2	IRS2		Q9Y4H2	Y4(Phospho)	309	33		3	751.6782	55.75	7753		2.14		2.33		2.73					
qVVESAyEVIk			Y239	Isoform 4 of L-lactate dehydrogenase A chain	LDHA	LDHA		P00338-4	Y7(Phospho)	274	32		3	601.6623	77.21	12417		1.00		0.88		0.96					
nLYSFcVSyVDSIQQMR			Y1070	Abl1 human spliceform 1a (myristoylated)	ABL1	Abl1		P00519	Y9(Phospho)	343	45		3	807.0413	83.27	13748		2.90		3.49		4.39					
kySLTVAVk			Y264	Abl1 human spliceform 1a (myristoylated)	ABL1	Abl1		P00519	Y2(Phospho)	420	26	2.31	3	592.6940	69.45	10718	10	67.11	99.49	54.43	56.00	34.36	29.36				
iNTASDGkLyVSSESR			Y185	Abl1 human spliceform 1a (myristoylated)	ABL1	Abl1		P00519	Y10(Phospho)	326	29	3.75	3	755.7221	66.64	10113	7	16.07	26.47	14.34	74.16	12.43	30.34				
lmTGDtyTAHAGAk			T392&Y393	Abl1 human spliceform 1a (myristoylated)	ABL1	Abl1		P00519	T6(Phospho); Y7(Phospho)	260	30		3	690.9859	54.24	7431		31.26		6.40		6.92					
lmTGDTytAHAGAk			Y393&T394	Abl1 human spliceform 1a (myristoylated)	ABL1	Abl1		P00519	Y7(Phospho); T8(Phospho)	309	30		3	690.9838	52.58	7078	2	10.39	2.19	9.42	1.38	11.24	4.47				
iNTAsDGkLyVSSESR			S180&Y185	Abl1 human spliceform 1a (myristoylated)	ABL1	Abl1		P00519	S5(Phospho);Y10(Phospho)	321	26		3	782.3778	68.39	10487		28.53		19.83		18.33					
ySLTVAVk			Y264	Abl1 human spliceform 1a (myristoylated)	ABL1	Abl1		P00519	Y1(Phospho)	248	28		2	709.9065	71.97	11268	9	34.48	82.25	57.23	57.89	35.28	74.04				
lGGGQYGEVyEGVWk			Y257	Abl1 human spliceform 1a (myristoylated)	ABL1	Abl1		P00519	Y10(Phospho)	476	79		2	1090.5454	80.66	13179	7	13.20	7.07	10.97	4.28	10.71	5.01				
nLySFcVSYVDSIQQMR			Y1064	Abl1 human spliceform 1a (myristoylated)	ABL1	Abl1		P00519	Y3(Phospho)	410	65		3	807.0380	84.32	13974	2	2.62	0.10	3.39	2.24	3.52	0.22				
hSWyHGPVSR			Y128	Abl1 human spliceform 1a (myristoylated)	ABL1	Abl1		P00519	Y4(Phospho)	286	33		3	512.2440	49.41	6398	4	5.31	1.69	7.00	9.09	5.93	1.64				
vLGyNHNGEWcEAQTk			Y93	Abl1 human spliceform 1a (myristoylated)	ABL1	Abl1		P00519	Y4(Phospho)	436	47		3	815.3854	68.50	10511	2	8.57	3.59	10.20	3.39	10.99	8.79				
nAAEyLLSSGINGSFLVR			Y139	Abl1 human spliceform 1a (myristoylated)	ABL1	Abl1		P00519	Y5(Phospho)	467	87		2	1110.5684	88.49	14867	2	23.96		24.35	2.15	17.82	7.03				
lGGGQyGEVYEGVWk			Y253	Abl1 human spliceform 1a (myristoylated)	ABL1	Abl1		P00519	Y6(Phospho)	353	48		3	727.3673	80.62	13168	2	41.62	48.52	58.65	73.71	42.21	53.35				
lGGGQyGEVyEGVWk			Y253&Y257	Abl1 human spliceform 1a (myristoylated)	ABL1	Abl1		P00519	Y6(Phospho); Y10(Phospho)	453	52		2	1130.5284	80.17	13071	3	20.36	11.91	18.45	8.79	11.93	5.02				
lMTGDTyTAHAGAk			Y393	Abl1 human spliceform 1a (myristoylated)	ABL1	Abl1		P00519	Y7(Phospho)	708	73		2	987.9915	56.81	7982	27	14.68	22.08	18.38	36.10	14.96	64.68				
gTPTAENPEyLGLDVPV			Y562	Isoform 3 of Receptor tyrosine-protein kinase erbB-2	ERBB2	ERBB2		P04626-3	Y10(Phospho)	296	41		2	1041.0095	84.55	14023		2.48		2.96		1.89					
lLDIDETEyHADGGk			Y191	Isoform 3 of Receptor tyrosine-protein kinase erbB-2	ERBB2	ERBB2		P04626-3	Y9(Phospho)	405	29		3	738.6960	77.39	12459		1.85		2.42		2.38					
iIQLLDDyPk			Y24	60S acidic ribosomal protein P0	RPLP0	RLA0		P05388	Y8(Phospho)	372	55		3	585.9965	84.73	14061		0.98		1.02		1.10					
gAySLSIR			Y185	Isoform 3 of Tyrosine-protein kinase Fyn	FYN	FYN		P06241-3	Y3(Phospho)	208	24		2	588.3048	63.73	9477		1.76		1.47		1.67					
lIEDNEyTAR			Y365	Isoform 3 of Tyrosine-protein kinase Fyn	FYN	FYN		P06241-3	Y7(Phospho)	233	49		3	511.5784	59.28	8518	14	1.72	0.44	1.89	0.53	1.91	0.58				
iGEGtyGVVYk			T14&Y15	Isoform 2 of Cyclin-dependent kinase 1	CDK1	CDK1		P06493-2	T5(Phospho); Y6(Phospho)	401	42		2	902.4409	76.04	12158	7	1.01	0.15	0.82	0.22	1.09	0.19				
iGEGTyGVVYk			Y15	Isoform 2 of Cyclin-dependent kinase 1	CDK1	CDK1		P06493-2	Y6(Phospho)	386	53		2	862.4574	74.38	11794	12	1.21	0.33	0.85	0.43	1.09	0.37				
sAySSySAPVSSSLSVR			Y40&Y43	Neurofilament light polypeptide	NEFL	NFL		P07196	Y3(Phospho); Y6(Phospho)	n/a		1.93	2	1068.9777	67.37	10270		6.54		7.84		2.00					
dLyDAGVk			Y199	Isoform 1 of Annexin A2	ANXA2	ANXA2		P07355-1	Y3(Phospho)	n/a		1.87	2	709.8746	61.92	9087		3.10		2.43		2.29					
vIEDNEyTAR			Y397	Isoform 2 of Tyrosine-protein kinase Lyn	LYN	LYN		P07948-2	Y7(Phospho)	371	44		2	759.8555	52.11	6978	3	1.12	0.40	1.07	0.24	0.97	0.56				
tTPSyVAFTDTER			Y41	Isoform 2 of Heat shock 70 kDa protein 1A/1B	HSPA8	HSP7C		P08107-2;P11142-2	Y5(Phospho)	301	24		2	898.9191	66.16	10007		11.31		8.99		14.47					
yIDQEELNk			Y276	Heat shock protein HSP 90-beta	HSP90AA1	HS90A		P08238;P07900-1	Y1(Phospho)	351	35		3	563.9550	65.02	9763	2	2.16	0.04	2.33	0.71	2.29	0.40				
iRyESLTDPSk			Y562	Heat shock protein HSP 90-beta	HSP90AA1	HS90A		P08238;P07900-1	Y3(Phospho)	n/a		3.2	3	616.3294	65.44	9853		2.55		3.15		2.45					
sVSPTTEmVSNESVDyR			Y1003	Isoform 1 of Hepatocyte growth factor receptor	MET	MET		P08581-1	Y16(Phospho)	252	45		3	742.6652	56.61	7937		1.28		1.74		1.96					
sINPDEAVAyGAAVQAAILSGDk			Y371	Isoform 2 of Heat shock cognate 71 kDa protein	HSPA8	HSP7C		P11142-2	Y10(Phospho)	553	78		3	933.4847	86.87	14519	2	11.72	13.29	11.77	5.27	11.07	5.33				
iTLDNAymEk			Y133	Isoform 3 of Pyruvate kinase isozymes M1/M2	PKM	KPYM		P14618-3	Y7(Phospho)	240	26		3	584.6278	69.08	10637		1.40		1.62		2.44					
aDGGAEyATYQTk			Y321	Isoform 3 of Membrane cofactor protein	CD46	MCP		P15529-16	Y7(Phospho)	260	33		3	638.3077	54.22	7427		2.13		1.77		3.51					
eHyVDLk			Y52	Isoform 1 of Nucleoside diphosphate kinase A	NME1	NDKA		P15531-1	Y3(Phospho)	300	30		3	481.2548	59.32	8527		0.77		1.02		1.06					
vQENDGkEPPPVVNyEEDAR			Y402	Isoform 2 of Tyrosine-protein kinase Fer	FER	FER		P16591-2	Y15(Phospho)	n/a		2.6	3	941.7914	61.39	8973		1.59		1.20		1.55					
sFLDSGyR			Y822	Isoform 1 of Vinculin	VCL	VINC		P18206-2	Y7(Phospho)	281	24		2	627.2927	67.89	10380	3	1.32	0.20	1.41	0.31	1.47	0.58				
vVQEYIDAFSDyANFk			Y798	Isoform 2 of Receptor-type tyrosine-protein phosphatase alpha	PTPRA	PTPRA		P18433-2	Y12(Phospho)	387	77		4	612.5541	89.06	14989	3	1.05	0.11	0.67	0.12	0.93	0.06				
hELQANcyEEVkDR			Y140	Cofilin-1	CFL1	COF1		P23528	Y8(Phospho)	375	21		3	777.0398	52.89	7145	2	2.13	0.02	2.84	0.03	2.95	0.44				
lAQyESk			Y126	Elongation factor 1-beta	EEF1B2	EF1B		P24534	Y4(Phospho)	247	21		2	688.8641	55.79	7761		2.22		1.90		2.09					
iADPEHDHTGFLTEyVATR			Y204	Isoform 2 of Mitogen-activated protein kinase 3	MAPK3	MK03		P27361-2	Y15(Phospho)	329	27		3	827.7290	76.81	12330		1.70		2.21		2.58					
vADPDHDHTGFLtEyVATR			T202&Y204	Isoform 2 of Mitogen-activated protein kinase 1	MAPK1	MK01		P28482-2	T13(Phospho); Y15(Phospho)	330	20		3	845.0369	78.13	12622		1.37		1.66		1.91					
vLEDDPEATyTTSGGk			Y772	Ephrin type-A receptor 2	EPHA2	EPHA2		P29317	Y10(Phospho)	322	27		3	741.0280	65.53	9871	2	0.93	0.01	1.60	0.00	0.93	0.04				
lLAQAEGEPcyIR			Y292	Non-receptor tyrosine-protein kinase TYK2	TYK2	TYK2		P29597	Y11(Phospho)	385	48		3	610.3004	77.15	12405	3	2.11	0.58	2.44	0.86	2.05	0.65				
vDPVDGVVLVDPDyLk			Y48	Isoform 1 of Beta-arrestin-2	ARRB2	ARRB2		P32121-1	Y14(Phospho)	493	58		3	761.0776	84.81	14078		1.19		1.11		1.10					
iAIyELLFk			Y12	40S ribosomal protein S10	RPS10	RS10		P46783	Y4(Phospho)	295	19		2	824.4806	88.36	14840		1.81		1.84		2.85					
vPDFSEyR			Y91	Cytochrome b-c1 complex subunit Rieske, mitochondrial	UQCRFS1	UCRI		P47985	Y7(Phospho)	n/a		2.35	2	661.3057	71.43	11149		1.78		5.86		5.16					
yYTPTISR			Y146	Proteasome subunit beta type-2	PSMB2	PSB2		P49721	Y1(Phospho)	n/a		2	2	655.3250	58.17	8274		1.34		1.25		1.41					
gEPNVSyIcSR			Y216	Isoform 1 of Glycogen synthase kinase-3 beta	GSK3B	GSK3B		P49841-1	Y7(Phospho)	266	47		3	530.9116	58.84	8420	40	1.29	0.46	1.29	0.57	1.32	0.54				
gEPNVSyIcsR			Y216&S219	Isoform 1 of Glycogen synthase kinase-3 beta	GSK3B	GSK3B		P49841-1	Y7(Phospho); S10(Phospho)	270	20		3	557.5666	58.15	8271	3	1.44	0.42	1.95	0.80	1.99	0.77				
aTENDIyNFFSPLNPVR			Y306	Heterogeneous nuclear ribonucleoprotein F	HNRNPF	HNRPF		P52597	Y7(Phospho)	421	61		3	769.3742	87.66	14689	2	9.75	4.16	11.27	7.30	3.94	0.18				
tTDGVyEGVAIGGDR			Y672	Isoform 2 of ATP-citrate synthase	ACLY	ACLY		P53396-2	Y6(Phospho)	295	52		3	606.9536	63.82	9499	3	1.02	0.44	1.44	0.41	1.49	0.44				
qADSEmTGyVVTR			Y185	Mitogen-activated protein kinase 12	MAPK12	MK12		P53778	Y9(Phospho)	228	43		3	594.6053	47.02	5884	2	2.19	1.63	2.30	1.82	2.17	1.63				
vLEDDPEAAyTTR			Y833	Isoform 3 of Ephrin type-A receptor 5	EPHA5	EPHA5		P54756-3	Y10(Phospho)	467	51		2	894.9158	63.06	9333	4	1.31	1.08	1.23	1.22	1.07	1.15				
dSyVGDEAQSk			Y53	Actin, cytoplasmic 1	ACTB	ACTB		P60709	Y3(Phospho)	232	47		3	579.6102	49.78	6478		1.54		2.35		2.20					
vLLPEyGGTk			Y76	10 kDa heat shock protein, mitochondrial	HSPE1	CH10		P61604	Y6(Phospho)	261	34		3	538.9697	74.71	11867	3	1.80	0.31	1.78	0.24	1.99	0.31				
eVSTyIk			Y177	Elongation factor 1-alpha 1	EEF1A1	EF1A1		P68104	Y5(Phospho)	180	20		2	689.3734	62.66	9246		1.38		1.73		1.92					

Supplementary Table 1

'Light' phosphotyrosine peptides

peptide - sequence			phospho site(s)	protein descriptions	gene name	synonyms		swissprot protein group accessions	phosphorylation modification	phosphoRS Score	Mascot Ion Score	Sequest XCorr	peptide charge	m/z [Th]	retention time (min)	# scan	# PSM	30 min 127/126 PP/Kin$^-$	STDEV	90 min 129/128 PP/Kin$^-$	STDEV	150 min 131/130 PP/Kin$^-$	STDEV	active kinase rate	STDEV	inactive kinase rate	STDEV
aATSDLEHyDk			**Y169**	**Isoform 1 of Nucleobindin-2**	**NUCB2**	**NUCB2**		P80303-1	Y9(Phospho)			2.19	3	596.6276	57.89	8215		2.18		2.52		2.41					
lGEGTyATVYk			**Y176**	**Isoform 1 of Cyclin-dependent kinase 16**	**CDK16**	**CDK16**		Q00536-1	Y6(Phospho)			1.99	2	870.4564	69.37	10699	2	1.16	0.23	1.34	0.13	1.18	0.01				
iDFYFDENPyFENk			**Y121**	**Isoform 3 of Protein SET**	**SET**	**SET**		Q01105-3	Y10(Phospho)	254	23		3	793.7039	84.12	13930		1.88		2.76		3.42					
gHEyTNIk			**Y542**	**Isoform 2 of Tyrosine-protein phosphatase non-receptor type 11**	**PTPN11**	**PTN11**		Q06124-2	Y4(Phospho)	338	43		3	500.5934	47.92	6080	3	1.24	0.17	1.31	0.32	1.34	0.06				
kPTyDPVSEDQDPLSSDFk			**Y518**	**Isoform 1 of Activated CDC42 kinase 1**	**TNK2**	**ACK1**		Q07912-1	Y4(Phospho)	518	50		3	979.1586	78.38	12678	4	1.32	0.10	1.56	0.37	1.40	0.19				
eTVyGcFQcSVDGQkyVR			**Y323&Y335**	**Pejvakin**	**DFNB59**	**PJVK**		Q0ZLH3	Y4(Phospho);Y16(Phospho)			2.59	4	704.3130	71.83	11237		19.85		9.40		12.96					
iVGSkPLyVALAQR			**Y364**	**Isoform 2 of Polyadenylate-binding protein 4**	**PABPC4**	**PABP4**		Q13310-2	Y8(Phospho)			2.68	3	685.0706	80.61	13166		12.53		12.09		10.00					
vDyVVVDQQk			**Y659**	**Isoform 1 of GRB2-associated-binding protein 1**	**GAB1**	**GAB1**		Q13480-1	Y3(Phospho)	277	50		3	577.6431	69.10	10643	2	1.65	0.03	1.86	0.36	1.72	0.01				
iYQyIQSR			**Y321**	**Isoform 2 of Dual specificity tyrosine-phosphorylation-regulated kinase 1A**	**DYRK1A**	**DYR1A**		Q13627-3	Y4(Phospho)	274	30		2	690.3497	49.00	6309	3	0.79	0.68	0.97	0.28	1.02	0.34				
lPSsPVyEDAASFk			**S381&Y384**	**Isoform 3 of Src substrate cortactin**	**CTTN**	**SRC8**		Q14247-3	S4(Phospho); Y7(Phospho)	379	40		2	1065.0076	80.42	13126	2	3.50	0.46	5.20	1.90	4.98	2.65				
gPVSGTEPEPVySmEAADYR			**Y416**	**Isoform 3 of Src substrate cortactin**	**CTTN**	**SRC8**		Q14247-3	Y12(Phospho)	292	17		3	827.3691	62.11	9126		5.65		5.43		7.72					
lPSSPVyEDAASFk			**Y384**	**Isoform 3 of Src substrate cortactin**	**CTTN**	**SRC8**		Q14247-3	Y7(Phospho)	239	20		3	683.6850	80.13	13062		3.78		8.20		7.55					
gFcyVEFDEVDSLk			**Y86**	**Isoform Short of Eukaryotic translation initiation factor 4H**	**EIF4H**	**IF4H**		Q15056-2	Y4(Phospho)	317	54		3	749.3569	84.42	13994		1.52		1.78		1.95					
aDQEGDEELyFHFk			**Y597**	**Isoform 2 of Ephrin type-A receptor 7**	**EPHA7**	**EPHA7**		Q15375-2	Y10(Phospho)	390	42		3	756.0198	79.73	12974		1.68		1.62		1.71					
vIEDDPEAVyTTTGGk			**Y791**	**Isoform 2 of Ephrin type-A receptor 7**	**EPHA7**	**EPHA7**		Q15375-2	Y10(Phospho)	461	62		3	745.0395	72.12	11301	8	1.20	0.26	1.23	0.43	1.11	0.27				
hTDDEMTGyVATR			**Y182**	**Isoform Mxi2 of Mitogen-activated protein kinase 14**	**MAPK14**	**MK14**		Q16539-3	Y9(Phospho)	346	46		3	602.2637	49.12	6334	3	3.96	1.63	1.94	0.76	1.67	1.67				
eATQPEPIyAESTk			**Y413**	**Tyrosine-protein kinase SgK223**	**SGK223**	**SG223**		Q86YV5	Y9(Phospho)	297	45		3	701.3536	57.13	8049	2	1.75	0.41	1.72	0.17	2.09	0.05				
aTTPPNQGRPDsPVyANLQELk			**S240&Y243**	**Isoform 3 of Rho GTPase-activating protein 12**	**ARHGAP12**	**RHG12**		Q8IWW6-3	S12(Phospho); Y15(Phospho)	314	28		4	754.3752	78.63	12733		2.09		2.35		2.59					
tLEPVkPPTVPNDymTSPAR			**Y213**	**Isoform 11 of Abl interactor 1**	**ABI1**	**ABI1**		Q8IZP0-11	Y14(Phospho)	279	21		3	923.1434	69.19	10663	3	2.88	0.23	2.72	0.50	2.46	0.88				
nTyNQTALDIVNQFTTSQASR			**Y253**	**Isoform 2 of Caskin-2**	**CASKIN2**	**CSKI2**		Q8WXE0-2	Y3(Phospho)	412	53		3	894.4305	85.98	14330		1.39		1.33		1.04					
vVAIyDYTk			**Y460**	**Isoform 3 of Abl interactor 2**	**ABI2**	**ABI2**		Q9NYB9-3	Y5(Phospho)	268	19		2	805.4348	77.99	12591		4.18		3.53		5.17					
vALENDDRSEEEkyTAVQR			**Y471**	**Isoform 2 of Ataxin-2**	**ATXN2**	**ATX2**		Q99700-2	Y14(Phospho)			1.94	4	698.3457	59.37	8538		12.64		7.67		8.28					
aNQLAEEIESSAQyk			**Y454**	**Isoform 2 of Ataxin-2**	**ATXN2**	**ATX2**		Q99700-2	Y14(Phospho)	489	56		3	740.3719	81.79	13428		8.73		8.50		10.81					
sAEEAPLySk			**Y389**	**Isoform 2 of Tyrosine-protein phosphatase non-receptor type 18**	**PTPN18**	**PTN18**		Q99952-2	Y8(Phospho)			2.56	3	544.9478	57.48	8127		3.54		3.97		3.25					
tyLGEEGk			**Y103**	**Isoform 2 of N-terminal Xaa-Pro-Lys N-methyltransferase 1**	**NTMT1**	**NTM1A**		Q9BV86-2	Y2(Phospho)			1.77	2	717.8672	55.88	7779		1.18		1.36		1.52					
vVDYSQFQEsDDADEDyGRDSGPPTk			**S19&Y26**	**Isoform 1 of Nuclear ubiquitous casein and cyclin-dependent kinase substrate 1**	**NUCKS1**	**NUCKS**		Q9H1E3-1	S10(Phospho); Y17(Phospho)	293	32		4	885.3792	72.13	11302		3.27		3.86		4.57					
vVDYSQFQESDDADEDyGRDSGPPTk			**Y26**	**Isoform 1 of Nuclear ubiquitous casein and cyclin-dependent kinase substrate 1**	**NUCKS1**	**NUCKS**		Q9H1E3-1	Y17(Phospho)	470	58		4	865.3880	71.20	11099	2	3.95	0.25	2.93	0.16	3.31	0.60				
aVcSTyLQSR			**Y361**	**Isoform 2 of Homeodomain-interacting protein kinase 2**	**HIPK2**	**HIPK2**		Q9H2X6-2	Y6(Phospho)	280	32		2	747.3541	52.83	7132	2	1.07	0.07	0.99	0.05	1.06	0.02				
yAAGVR			**Y508**	**Polyadenylate-binding protein 3**	**PABPC3**	**PABP3**		Q9H361	Y1(Phospho)			1.76	2	473.2414	40.56	4518		10.12		5.44		3.45					
vPSEGAyDIILPR			**Y399**	**Isoform 1 of G-protein coupled receptor family C group 5 member C**	**GPRC5C**	**GPC5C**		Q9NQ84-1	Y7(Phospho)	342	63		3	580.3043	81.41	13342		1.62		2.10		2.38					
nEEENIySVPHDSTQGk			**Y1105**	**Rho GTPase-activating protein 35**	**ARHGAP35**	**RHG35**		Q9NRY4	Y7(Phospho)	303	47		3	829.0604	57.20	8064		1.65		1.80		2.62					
sPSScNDLyATVk			**Y359**	**Phosphoprotein associated with glycosphingolipid-enriched microdomains 1**	**PAG1**	**PHAG1**		Q9NWQ8	Y9(Phospho)	487	58		2	990.4821	63.07	9334	4	1.34	0.29	1.65	0.37	1.38	0.37				
sVDGDQGLGmEGPyEVLk			**Y163**	**Phosphoprotein associated with glycosphingolipid-enriched microdomains 1**	**PAG1**	**PHAG1**		Q9NWQ8	Y14(Phospho)	338	38		3	816.7300	76.98	12367		2.58		2.39		1.59					
sGQSLTVPESTyTSIQGDPQR			**Y341**	**Phosphoprotein associated with glycosphingolipid-enriched microdomains 1**	**PAG1**	**PHAG1**		Q9NWQ8	Y12(Phospho)	363	65		3	854.0767	67.93	10389	4	1.87	0.77	1.61	0.59	2.24	0.24				
tPNSTLPPAGRPSEEPEPDyEAIQTLNREEEk			**Y387**	**Phosphoprotein associated with glycosphingolipid-enriched microdomains 1**	**PAG1**	**PHAG1**		Q9NWQ8	Y20(Phospho)			3.71	4	1034.0125	78.18	12633		2.91		1.66		1.25					
eNDyESISDLQQGR			**Y417**	**Phosphoprotein associated with glycosphingolipid-enriched microdomains 1**	**PAG1**	**PHAG1**		Q9NWQ8	Y4(Phospho)	411	60		2	981.9360	68.03	10410	2	1.42	0.03	1.49	0.55	1.68	0.87				
aEFAEyAsVDR			**Y227&S229**	**Phosphoprotein associated with glycosphingolipid-enriched microdomains 1**	**PAG1**	**PHAG1**		Q9NWQ8	Y6(Phospho); S8(Phospho)	344	51		2	823.8384	63.93	9522		2.89		2.98		3.21					
aEFAEyASVDR			**Y227**	**Phosphoprotein associated with glycosphingolipid-enriched microdomains 1**	**PAG1**	**PHAG1**		Q9NWQ8	Y6(Phospho)	296	52		3	522.9063	65.34	9831	6	1.43	0.35	1.26	0.36	1.33	0.21				
rEEPEALyAAVNk			**Y968**	**Isoform 4 of Intersectin-2**	**ITSN2**	**ITSN2**		Q9NZM3-4	Y8(Phospho)	493	51		3	676.6914	70.59	10964	5	1.66	0.47	2.05	0.37	1.96	0.72				

Supplementary Table 1

phosphotyrosine peptides

peptide - sequence	phospho-site	protein descriptions	gene name	synonyms	swissprot protein group accessions	phosphorylation modification	phosphoRS Binomial Peptide Score	Mascot Ion Score	SequestXCorr	peptide charge	m/z [Th]	retention time (min)	# PSM	30 min 127/126 PP/Kin⁻	CV (%)	90 min 129/128 PP/Kin⁻	CV (%)	150 min 131/130 PP/Kin⁻	CV (%)
AYTNFDAER	Y30	Putative annexin A2-like protein	ANXA2P2	AXA2L	A6NMY6	Y2(Phospho)	146	23		2	698.311	50.57	1	1.17		1.45		1.42	
EYVDPNNIFGNR	Y645	Alkyldihydroxyacetonephosphate synthase, peroxisomal	AGPS	ADAS	O00116	Y2(Phospho)	222	55		2	873.907	69.10	1	9.48		20.05		12.18	
GHFFVEDQIYCEK	Y306	PDZ and LIM domain protein 1	PDLIM1	PDLI1	O00151	Y10(Phospho)	304	39		3	737.351	70.96	1	3.53		8.65		11.31	
VIYDFIEK	Y256	Neural Wiskott-Aldrich syndrome protein	WASL	WASL	O00401	Y3(Phospho)	164	25		2	782.927	78.34	1	0.84		0.97		0.79	
SDYDGIGSR	Y104	Isoform 2 of ATP-dependent RNA helicase DDX3X	DDX3X	DDX3X	O00571-2	Y3(Phospho)	n/a	20		2	639.782	44.59	1	2.52		7.68		8.23	
SEYAPSEK	Y11	Isoform 2 of Phytanoyl-CoA dioxygenase, peroxisomal	PHYH	PAHX	O14832-2	Y3(Phospho)	n/a	26		2	724.857	43.15	1	4.37		10.04		13.86	
VCEPCYEQLNR	Y216	Isoform 2 of Hepatocyte growth factor-regulated tyrosine kinase substrate	HGS	HGS	O14964-2	Y6(Phospho)	174	34		2	888.886	52.35	1	3.88		8.62		9.78	
VEGDNIYVR	Y623	Isoform 1 of Band 4.1-like protein 2	EPB41L2	E41L2	O43491-1	Y7(Phospho)	209	39		2	687.337	57.91	1	1.01		1.19		1.02	
KQTETEMLYGSAPR	Y464	Isoform 3 of Protein regulator of cytokinesis 1	PRC1	PRC1	O43663-3	Y9(Phospho)	107	26		3	722.362	50.66	1	3.13		6.02		8.25	
ADGYNQPDSK	Y535	Isoform 2 of Heterogeneous nuclear ribonucleoprotein Q	SYNCRIP	HNRPQ	O60506-2	Y4(Phospho)	n/a	24		2	816.887	39.61	1	5.90		21.49		13.13	
LVNEAPVYSVYSK	Y371	Isoform 1 of Signal transducing adapter molecule 2	STAM2	STAM2	O75886-1	Y8(Phospho)	210	43		2	1004.034	66.73	1	5.23		16.04		15.68	
VGVGTCGIADKPMTQYQDTSK	Y224	Survival of motor neuron-related-splicing factor 30	SMNDC1	SPF30	O75940	Y16(Phospho)	198	59		4	760.634	57.46	5	12.50	34.0	21.50	41.5	17.72	8.7
DDEEADAIYAALDK	Y105	Isoform 2 of Pre-mRNA-processing factor 6	PRPF6	PRP6	O94906-2	Y9(Phospho)	214	41		2	692.999	75.75	1	1.84		3.53		3.73	
LEEGGPVYSPPAEVVVK	Y141	Isoform 2 of LETM1 and EF-hand domain-containing protein 1, mitochondrial	LETM1	LETM1	O95202-2	Y8(Phospho)	n/a	42	4.37	3	770.079	66.60	1	4.48		8.05		5.04	
SESVVYADIR	Y263	Isoform 5 of Myelin protein zero-like protein 1	MPZL1	MPZL1	O95297-5	Y6(Phospho)	110	56		2	724.356	60.69	2	1.23	19.5	1.78	25.2	1.41	34.4
QVVESAYEVIK	Y293	Isoform 4 of L-lactate dehydrogenase A chain	LDHA	LDHA	P00338-4	Y7(Phospho)	172	40		3	601.662	67.08	1	1.09		1.45		1.36	
LGGGQYGEVYEGVWK	Y253&Y257	Isoform IA of Tyrosine-protein kinase ABL1	ABL1	ABL1	P00519-1	Y6(Phospho); Y10(Phospho)	245	65		2	1130.530	70.93	2	44.80	99.4	17.98	11.8	12.02	6.9
INTASDGKLYVSSESR	S180&Y185	Isoform IA of Tyrosine-protein kinase ABL1	ABL1	ABL1	P00519-1	S5(Phospho); Y10(Phospho)	99	34		2	1173.064	61.31	1	27.57		16.13		14.51	
YSLTVAVK	Y264	Isoform IA of Tyrosine-protein kinase ABL1	ABL1	ABL1	P00519-1	Y1(Phospho)	153	36		2	709.907	64.21	3	47.94	129.1	14.50	20.5	10.79	1.9
VYELMR	Y449	Isoform IA of Tyrosine-protein kinase ABL1	ABL1	ABL1	P00519-1	Y2(Phospho)	77	21		2	568.274	54.13	1	27.48		12.33		14.31	
LGGGQYGEVYEGVWK	Y257	Isoform IA of Tyrosine-protein kinase ABL1	ABL1	ABL1	P00519-1	Y10(Phospho)	245	71		2	1090.547	71.01	6	28.89	215.4	11.59	13.0	12.57	23.5
VLGYNHNGEWCEAQTK	Y93	Isoform IA of Tyrosine-protein kinase ABL1	ABL1	ABL1	P00519-1	Y4(Phospho)	n/a	20	3.51	3	815.391	65.08	1	14.16		16.40		11.58	
LYVSSESR	Y185	Isoform IA of Tyrosine-protein kinase ABL1	ABL1	ABL1	P00519-1	Y2(Phospho)	n/a	30		2	625.305	52.70	2	44.74	18.9	12.39	8.9	12.64	10.1
KYSLTVAVK	Y264	Isoform IA of Tyrosine-protein kinase ABL1	ABL1	ABL1	P00519-1	Y2(Phospho)	n/a	34	3.69	3	592.693	63.89	3	31.16	71.1	9.74	17.4	10.85	1.6
LMTGDTYTAHAGAK	Y393&T394	Isoform IA of Tyrosine-protein kinase ABL1	ABL1	ABL1	P00519-1	Y7(Phospho); T8(Phospho)	n/a	32		3	690.985	50.13	2	9.91	51.1	9.05	70.0	9.27	31.1
LMTGDTYTAHAGAK	T392&Y393	Isoform IA of Tyrosine-protein kinase ABL1	ABL1	ABL1	P00519-1	T6(Phospho); Y7(Phospho)	n/a	42		3	685.652	55.00	1	9.06		8.67		8.94	
INTASDGKLYVSSESR	Y185	Isoform IA of Tyrosine-protein kinase ABL1	ABL1	ABL1	P00519-1	Y10(Phospho)	n/a	26		3	755.723	60.07	1	16.77		7.85		8.69	
DIYETDYYRK	Y1173,Y1177&Y1178	Isoform Short of Insulin receptor	INSR	INSR	P06213-2	Y3(Phospho); Y7(Phospho); Y8(Phospho)	148	21		3	688.624	50.69	1	0.87		1.10		0.74	
DIYETDYYRK	Y1173 & Y1177	Isoform Short of Insulin receptor	INSR	INSR	P06213-2	Y3(Phospho); Y7(Phospho)	n/a	28	2.56	3	661.968	55.17	2	0.87	2.5	1.09	6.2	0.82	2.4
DGSLNQSSGYR	Y28	Isoform 3 of Tyrosine-protein kinase Fyn	FYN	FYN	P06241-3	Y10(Phospho)	130	41		2	746.835	40.66	1	1.36		1.22		0.95	
LIEDNEYTAR	Y365	Isoform 3 of Tyrosine-protein kinase Fyn	FYN	FYN	P06241-3	Y7(Phospho)	178	45	3.95	3	511.578	55.60	20	1.03	13.5	1.11	19.3	1.01	24.3
IGEGTYGVVYK	Y15	Isoform 2 of Cyclin-dependent kinase 1	CDK1	CDK1	P06493-2	Y6(Phospho)	188	62		2	862.458	64.20	14	0.88	9.0	1.11	24.2	0.73	21.6
IGEGTYGVVYK	T14&Y15	Isoform 2 of Cyclin-dependent kinase 1	CDK1	CDK1	P06493-2	T5(Phospho); Y6(Phospho)	156	38	3.86	2	902.441	65.83	12	0.89	11.4	1.03	16.6	0.98	28.2
AAVPSGASTGIYEALELR	Y44	Isoform alpha-enolase of Alpha-enolase	ENO1	ENOA	P06733-1	Y12(Phospho)	276	81		2	1057.543	76.94	2	4.74	26.0	14.68		11.68	24.0
YISPDQLADLYK	S272&Y280	Isoform alpha-enolase of Alpha-enolase	ENO1	ENOA	P06733-1	S3(Phospho); Y11(Phospho)	214	40		3	682.000	72.82	1	3.34		8.86		10.34	
YISPDQLADLYK	Y280	Isoform alpha-enolase of Alpha-enolase	ENO1	ENOA	P06733-1	Y11(Phospho)	n/a	31		3	655.345	72.46	1	3.16		10.82		11.34	
MVVESAYEVIK	Y240	L-lactate dehydrogenase B chain	LDHB	LDHB	P07195	Y7(Phospho)	167	28		3	607.988	66.48	1	1.82		2.40		2.03	
VIEDNEYTAR	Y397	Isoform 1 of Tyrosine-protein kinase Lyn	LYN	LYN	P07948-1	Y7(Phospho)	140	43	3.32	2	759.856	48.49	5	0.94	8.6	1.09	7.6	1.01	25.9
EDQTEYLEER	Y192	Heat shock protein HSP 90-beta	HSP90AA1	HS90A	P08238;P07900-1	Y6(Phospho)	140	39		2	810.853	49.13	1	0.96		1.22		1.03	
YIDQEELNK	Y276	Heat shock protein HSP 90-beta	HSP90AA1	HS90A	P08238;P07900-1	Y1(Phospho)	161	38		2	845.429	59.28	2	1.34	15.1	2.04	21.4	2.14	28.3
SLYASSPGGVYATR	Y61	Vimentin	VIM	VIME	P08670	Y11(Phospho)	154	29		2	869.425	56.22	1	2.08		4.31		5.72	
LYDAYELK	Y94	Annexin A5	ANXA5	ANXA5	P08758	Y5(Phospho)	132	25		2	776.907	67.51	1	0.95		1.27		1.00	
ASCLYGQLPK	Y50	Glutathione S-transferase P	GSTP1	GSTP1	P09211	Y5(Phospho)	167	28		3	558.961	62.88	1	2.57		6.48		7.07	
SSGPYGGGGQYFAKPR	Y347	Isoform 2 of Heterogeneous nuclear ribonucleoprotein A1	HNRNPA1	ROA1	P09651-3	Y11(Phospho)	231	46	4.38	2	1084.042	56.25	10	18.30	32.6	21.52	17.1	13.89	8.4
SSGPYGGGGQYFAKPR	Y341&Y347	Isoform 2 of Heterogeneous nuclear ribonucleoprotein A1	HNRNPA1	ROA1	P09651-3	Y5(Phospho); Y11(Phospho)	157	38		2	1124.024	53.12	1	5.53		14.80	67.2	9.52	67.0
LDGENIYIR	Y660	Isoform 7 of Protein 4.1	EPB41	41	P11171-1	Y7(Phospho)	148	44		2	701.352	67.51	1	1.09		1.95		1.59	
ITLDNAYMEK	Y148	Isoform 3 of Pyruvate kinase PKM	PKM	KPYM	P14618-3	Y7(Phospho)	n/a	27		3	584.628	62.08	1	1.90		3.73		3.51	
DISTNYYASQK	Y677	Endoplasmin	HSP90B1	ENPL	P14625	Y6(Phospho)	222	48		2	914.451	55.84	1	2.85		5.57		5.81	
ADGGAEYATYQTK	Y384	Isoform 3 of Membrane cofactor protein	CD46	MCP	P15529-16	Y7(Phospho)	205	49		2	956.959	49.61	2	1.30	18.1	1.98	9.9	1.93	17.6
SHYEEGPGK	Y19	Cytochrome c oxidase subunit 7C, mitochondrial	COX7C	COX7C	P15954	Y3(Phospho)	194	28		2	771.374	40.00	3	3.34	3.3	7.97	29.4	13.09	40.9
ETVYCIGQR	Y339	Isoform 2 of Cytoplasmic protein NCK1	NCK1	NCK1	P16333-2	Y4(Phospho)	136	24		2	717.836	50.91	1	2.71		5.77		5.28	
QEDGGVYSSSGLK	Y714	Isoform 1 of Tyrosine-protein kinase Fer	FER	FER	P16591-1	Y7(Phospho)	248	56		2	932.959	51.09	1	0.99		1.45		0.88	
VQENDGKEPPPVVNYEEDAR	Y402	Isoform 1 of Tyrosine-protein kinase Fer	FER	FER	P16591-1	Y15(Phospho)	210	39		3	941.792	55.31	2	0.86	2.7	1.03	3.3	0.75	16.2
SFLDSGYR	Y822	Isoform 1 of Vinculin	VCL	VINC	P18206-2	Y7(Phospho)	127	35		2	627.292	62.04	2	0.87	6.3	1.29	7.0	1.08	13.7
VVQEYIDAFSDYANFK	Y798	Isoform 2 of Receptor-type tyrosine-protein phosphatase alpha	PTPRA	PTPRA	P18433-2	Y12(Phospho)	280	45		3	816.405	84.67	1	0.88		0.98		0.69	
IGTAEPDYGALYEGR	Y771	Isoform 1 of 1-phosphatidylinositol 4,5-bisphosphate phosphodiesterase gamma-1	PLCG1	PLCG1	P19174-1	Y8(Phospho)	186	38		2	960.952	63.78	1	2.65		3.93		3.19	
EVYELLDSPGK	Y22	Isoform 1 of Multifunctional protein ADE2	PAICS	PUR6	P22234-1	Y3(Phospho)	196	41		2	894.467	70.83	2	5.96	3.7	10.07	10.6	7.99	8.0
AENYDIPSADR	Y873	Ubiquitin-like modifier-activating enzyme 1	UBA1	UBA1	P22314	Y4(Phospho)	117	34		2	780.351	49.30	1	9.94		17.83		16.84	
NMGGPYGGGNYGPGGSGGSGGYGGR	Y336	Isoform A2 of Heterogeneous nuclear ribonucleoproteins A2/B1	HNRNPA2B1	ROA2	P22626-2	Y11(Phospho)	156	64		3	839.015	46.12	1	2.60		6.62		6.62	
IPLPGAEMLEEEPLYVNAK	Y266	Isoform Short of Nuclear transcription factor Y subunit alpha	NFYA	NFYA	P23511-2	Y15(Phospho)	287	60		4	667.601	76.55	2	13.99	74.5	14.72	51.5	9.15	17.3
EILVGDVGQTVDDPYATFVK	Y68	Cofilin-1	CFL1	COF1	P23528	Y15(Phospho)	271	72		3	902.137	75.33	1	4.88		11.25		7.42	
MIYASSK	Y117	Cofilin-1	CFL1	COF1	P23528	Y3(Phospho)	n/a	18		2	669.350	53.67	1	22.22		32.83		9.60	
HELQANCYEEVKDR	Y140	Cofilin-1	CFL1	COF1	P23528	Y8(Phospho)	n/a	24	3.89	3	777.040	51.67	3	3.75	9.8	6.06	14.1	5.85	8.4
DVIEEYFK	Y127	40S ribosomal protein S12	RPS12	RS12	P25398	Y6(Phospho)	n/a	24		2	790.904	70.52	1	2.74		4.37		3.83	
LELAQYR	Y440	Isoform 2 of ATP synthase subunit alpha, mitochondrial	ATP5A1	ATPA	P25705-2	Y6(Phospho)	n/a	24		2	601.313	61.47	1	5.10		10.57		10.73	
QTIDNSQGAYQEAFDISK	Y149	14-3-3 protein theta	YWHAQ	1433T	P27348	Y10(Phospho)	233	56		3	851.749	66.46	1	3.44		4.83		3.08	
QSPEDVYFSK	Y575	Ephrin type-A receptor 2	EPHA2	EPHA2	P29317	Y7(Phospho)	188	35		2	869.429	61.38	2	1.30	33.9	1.69	47.7	1.56	79.4
VLEDDPEAAYTTR	Y779	Isoform 1 of Ephrin type-A receptor 3	EPHA3	EPHA3	P29320-1	Y10(Phospho)	202	47		2	894.917	58.51	4	0.98	2.8	1.21	9.7	0.90	9.2
LLAQAEGEPCYIR	Y292	Non-receptor tyrosine-protein kinase TYK2	TYK2	TYK2	P29597	Y11(Phospho)	200	49		2	914.947	67.23	3	1.08	20.6	1.27	6.0	1.04	6.6
RLAPEYEAAATR	Y677	Protein disulfide-isomerase A3	PDIA3	PDIA3	P30101	Y6(Phospho)	179	58		3	552.948	48.57	4	3.51	45.5	10.29	54.9	11.68	29.2

Supplementary Table 2

phosphotyrosine peptides

peptide - sequence	phospho-site	protein descriptions	gene name	synonyms	swissprot protein group accessions	phosphorylation modification	phosphoRS Binomial Peptide Score	Mascot Ion Score	SequestXCorr	peptide charge	m/z [Th]	retention time (min)	# PSM	30 min 127/126 PP/Kin⁻	CV (%)	90 min 129/128 PP/Kin⁻	CV (%)	150 min 131/130 PP/Kin⁻	CV (%)
DGMDNQGGYGSVGR	Y296	Isoform 4 of Heterogeneous nuclear ribonucleoprotein H3	HNRNPH3	HNRH3	P31942-4	Y9(Phospho)	156	49		2	861.361	43.00	1	13.35		34.69		15.56	
GPAYGLSR	Y29	Transgelin-2	TAGLN2	TAGL2	P37802	Y4(Phospho)	130	33		2	565.284	46.03	1	6.92		14.31		14.06	
RVPNAYDK	Y251	Isoform Crk-II of Adapter molecule crk	CRK	CRK	P46108-1	Y6(Phospho)	141	32		3	500.938	44.48	1	5.13		12.49		11.75	
IAIYELLFK	Y12	40S ribosomal protein S10	RPS10	RS10	P46783	Y4(Phospho)	186	30		2	824.482	83.99	2	4.93	5.8	5.88	4.3	4.03	3.3
VPDFSEYR	Y7	Cytochrome b-c1 complex subunit Rieske, mitochondrial	UQCRFS1	UCRI	P47985	Y7(Phospho)	99	29		2	661.305	63.56	2	6.73	30.2	18.41	61.1	13.70	9.4
LDVTSVEDYK	Y274	Isoform 2 of T-complex protein 1 subunit epsilon	CCT5	TCPE	P48643-2	Y9(Phospho)	154	39		2	853.936	66.91	1	1.86		4.38		4.69	
GITINAAHVEYSTAAR	Y115	Elongation factor Tu, mitochondrial	TUFM	EFTU	P49411	Y11(Phospho)	243	40		3	661.669	61.28	1	2.86		4.04		4.55	
NYEEIAK	Y135	Transmembrane emp24 domain-containing protein 10	TMED10	TMEDA	P49755	Y2(Phospho)	n/a	24		2	702.863	51.98	1	1.68		1.88		1.28	
GEPNVSYICSR	Y279	Isoform 1 of Glycogen synthase kinase-3 beta	GSK3B	GSK3B	P49841-1	Y7(Phospho)	207	51		2	795.863	54.96	19	0.99	11.7	1.17	19.3	0.98	25.6
SDSYVELSQYR	Y14	Isoform 3 of Nuclear cap-binding protein subunit 2	NCBP2	NCBP2	P52298-3	Y4(Phospho)	144	32		2	828.380	61.47	1	5.69		9.65		7.26	
QADSEMTGYVVTR	Y185	Isoform 2 of Mitogen-activated protein kinase 12	MAPK12	MK12	P53778-2	Y9(Phospho)	133	38		2	891.403	43.79	1	0.98		1.15		0.68	
EENPYAR	Y174	Pituitary tumor-transforming gene 1 protein-interacting protein	PTTG1IP	PTTG	P53801	Y5(Phospho)	145	26		2	594.268	36.45	2	1.11	1.1	1.01	22.7	1.99	3.8
AADEEAFEDNSEEYIRR	Y369	Isoform 4 of Exportin-2	CSE1L	XPO2	P55060-4	Y14(Phospho)	231	38		3	785.010	59.57	1	1.51		1.72		1.54	
LSHSDEKPYQCPVCQQR	Y307	Isoform 3 of Myc-associated zinc finger protein	MAZ	MAZ	P56270-3	Y9(Phospho)	n/a	26	4.42	3	890.758	47.00	3	9.79	43.1	15.03	23.7	12.77	13.7
RLEENDDDAYLNSPWADNTALK	Y264	Uncharacterized protein C21orf59	C21orf59	CU059	P57076	Y10(Phospho)	304	86		3	1030.162	70.84	1	4.03		11.46		10.98	
GYSFTTTAER	Y198	Actin, cytoplasmic 1	ACTB	ACTB	P60709	Y2(Phospho)	n/a	26		2	721.332	51.43	1	1.27		2.06		2.27	
VLLPEYGGTK	Y76	10 kDa heat shock protein, mitochondrial	HSPE1	CH10	P61604	Y6(Phospho)	141	27		2	807.950	65.30	1	1.01		1.36		1.23	
GGDLMAYDRR	Y323	Isoform 3 of Heterogeneous nuclear ribonucleoprotein K	HNRNPK	HNRPK	P61978-3	Y7(Phospho)	n/a	25		3	493.560	42.39	2	5.33	3.3	7.97	19.8	10.54	44.0
VETFSGVYK	Y177	40S ribosomal protein S7	RPS7	RS7	P62081	Y8(Phospho)	192	34		2	784.413	63.42	3	6.89	90.9	16.08	78.3	15.43	11.2
KQEGTPEGLYL	Y439	26S protease regulatory subunit 4	PSMC1	PRS4	P62191	Y10(Phospho)	n/a	36		2	886.966	69.50	1	4.54		7.32		7.97	
GHQQLYWSHPR	Y7	Isoform 1 of 40S ribosomal protein S29	RPS29	RS29	P62273-1	Y6(Phospho)	226	35		3	573.277	50.71	9	2.54	13.2	5.63	37.0	6.21	42.3
TSEVNCYR	Y159	Cellular nucleic acid-binding protein	CNBP	CNBP	P62633	Y7(Phospho)	126	29		2	669.292	39.40	2	8.09	140.5	24.61	18.3	15.70	8.6
EQCCYNCGKPGHLAR	Y99	Cellular nucleic acid-binding protein	CNBP	CNBP	P62633	Y5(Phospho)	234	29	3.94	4	597.776	44.83	10	12.69	59.5	25.87	56.2	15.12	11.7
CYSCGEFGHIQK	Y120	Cellular nucleic acid-binding protein	CNBP	CNBP	P62633	Y2(Phospho)	n/a	25		3	675.310	53.36	2	5.18	22.2	15.58	21.2	14.01	5.6
DCDLQEDACYNCGR	Y75	Isoform 2 of Cellular nucleic acid-binding protein	CNBP	CNBP	P62633-2	Y10(Phospho)	381	78		3	695.596	47.70	1	6.55		34.90	44.8	16.83	9.5
DCDLQEDEACYNCGR	Y76	Isoform 4 of Cellular nucleic acid-binding protein	CNBP	CNBP	P62633-4	Y11(Phospho)	311	61		2	1107.411	48.36	1	3.73		32.04	39.1	16.68	18.3
DCDLQEDVEACYNCGR	Y77	Isoform 5 of Cellular nucleic acid-binding protein	CNBP	CNBP	P62633-5	Y12(Phospho)	285	59		2	1156.946	61.27	2	4.50	150.6	10.75	192.1	9.16	134.0
LAPDYDALDVANK	Y144	60S ribosomal protein L23a	RPL23A	RL23A	P62750	Y5(Phospho)	264	52		2	972.002	68.20	3	3.51	52.2	11.02	3.4	11.02	5.9
EEAAEYAK	Y209	40S ribosomal protein S6	RPS6	RS6	P62753	Y6(Phospho)	n/a	34		2	724.857	43.77	1	9.37		11.13		7.54	
NLQYYDISAK	Y146	GTP-binding nuclear protein Ran	RAN	RAN	P62826	Y4(Phospho)	152	34		2	876.953	63.90	1	2.51		5.05		4.46	
NLQYYDISAK	Y146&Y147	GTP-binding nuclear protein Ran	RAN	RAN	P62826	Y4(Phospho); Y5(Phospho)	n/a		3.96	2	916.936	62.18	1	2.83		6.24		5.09	
AQVIYTR	Y109	40S ribosomal protein S25	RPS25	RS25	P62851	Y5(Phospho)	105	22		2	580.307	49.03	2	4.26	24.4	15.68	40.7	17.48	20.4
EEDGRYRDPTTVTTLR	Y153	Isoform 1 of Serine/threonine-protein phosphatase 2A 55 kDa regulatory subunit B alph	PPP2R2A	2ABA	P63151-1	Y6(Phospho)	n/a	24	3.80	3	740.029	50.44	6	4.42	19.0	7.54	26.6	10.35	37.6
YRDPTTVTTLR	Y153	Isoform 1 of Serine/threonine-protein phosphatase 2A 55 kDa regulatory subunit B alph	PPP2R2A	2ABA	P63151-1	Y1(Phospho)	n/a	28		3	544.617	51.29	2	2.96	19.3	5.35	18.7	8.31	13.8
TYAICGAIR	Y53	40S ribosomal protein S21	RPS21	RS21	P63220	Y2(Phospho)	167	27		2	667.331	59.96	1	10.99		17.71		15.83	
DETNYGIPQR	Y52	Guanine nucleotide-binding protein subunit beta-2-like 1	GNB2L1	GBLP	P63244	Y5(Phospho)	139	33		2	751.348	47.31	1	6.06		11.19		10.04	
STTTGHLIYK	Y29	Elongation factor 1-alpha 1	EEF1A1	EF1A1	P68104	Y9(Phospho)	250	50		2	829.950	55.94	9	6.73	51.2	17.56	31.8	12.94	4.6
LPLQDVYK	Y254	Elongation factor 1-alpha 1	EEF1A1	EF1A1	P68104	Y7(Phospho)	196	33	3.27	3	505.286	67.68	2	10.92	107.8	25.57	45.2	16.73	4.5
EHALLAYTLGVK	Y141	Elongation factor 1-alpha 1	EEF1A1	EF1A1	P68104	Y7(Phospho)	201	30		3	618.350	75.98	1	1.07		1.49		1.28	
DSLYAQGK	Y34	60S ribosomal protein L36a	RPL36A	RL36A	P83881	Y4(Phospho)	153	32		2	710.368	49.28	1	8.43		14.19		13.93	
SGDSEVYQLGDVSQK	Y73	Single-stranded DNA-binding protein, mitochondrial	SSBP1	SSBP	Q04837	Y7(Phospho)	233	57		2	1075.526	63.76	2	4.76	83.0	10.34	74.1	9.26	69.0
SDSASSEPVGIYQGFEK	Y313	Isoform 1 of Protein kinase C delta type	PRKCD	KPCD	Q05655-1	Y12(Phospho)	n/a		3.69	3	780.378	64.32	1	3.40		4.79		4.74	
IQNTGDYYDLYGGEK	Y63	Isoform 2 of Tyrosine-protein phosphatase non-receptor type 11	PTPN11	PTN11	Q06124-2	Y7(Phospho)	277	64	3.61	2	1137.539	66.25	3	1.64	89.8	3.33	81.5	2.94	300.9
VYENVGLMQQQK	Y584	Isoform 2 of Tyrosine-protein phosphatase non-receptor type 11	PTPN11	PTN11	Q06124-2	Y2(Phospho)	223	37		3	659.009	62.95	1	4.29		8.34		7.09	
GHEYTNIK	Y546	Isoform 2 of Tyrosine-protein phosphatase non-receptor type 11	PTPN11	PTN11	Q06124-2	Y4(Phospho)	n/a	22		3	500.594	45.04	2	1.62	33.9	2.24	30.8	2.05	21.7
ALPQNDDHYVMQEHR	Y284	Isoform 1 of Activated CDC42 kinase 1	TNK2	ACK1	Q07912-1	Y9(Phospho)	n/a	39		3	726.661	47.15	1	1.80		2.77		2.62	
EDIYSGGGGGGSR	Y180	Heterogeneous nuclear ribonucleoprotein A0	HNRNPA0	ROA0	Q13151	Y4(Phospho)	170	61		2	760.832	40.34	1	16.10		32.79		10.95	
VFPGSTTEDYNLIVIER	Y517	Isoform 2 of Transcription intermediary factor 1-beta	TRIM28	TIF1B	Q13263-2	Y10(Phospho)	210	55		3	754.715	76.15	1	4.40		3.35		3.35	
TVYCNVHK	Y208	Isoform 2 of Transcription intermediary factor 1-beta	TRIM28	TIF1B	Q13263-2	Y3(Phospho)	n/a	25		3	520.266	45.21	1	2.45		4.85		4.22	
KSLDSDESEDEEDDYQQK	S63&Y70	28 kDa heat- and acid-stable phosphoprotein	PDAP1	HAP28	Q13442	S8(Phospho); Y15(Phospho)	279	73		3	1003.103	51.12	2	1.71	9.8	3.83	5.7	3.42	8.1
KSLDSDESEDEEDDYQQK	S60&;63&Y70	28 kDa heat- and acid-stable phosphoprotein	PDAP1	HAP28	Q13442	S5(Phospho); S8(Phospho); Y15(Phospho)	124	25		3	1029.759	53.02	1	1.34		1.43		2.45	
LCDFGSASHVADNDITPYLVSR	Y849	Serine/threonine-protein kinase PRP4 homolog	PRPF4B	PRP4B	Q13523	Y18(Phospho)	219	58		3	916.096	73.58	2	1.18	5.9	1.42	15.6	1.58	49.3
IYQYIQSR	Y4	Isoform 2 of Dual specificity tyrosine-phosphorylation-regulated kinase 1A	DYRK1A	DYR1A	Q13627-3	Y4(Phospho)	123	28	3.59	2	690.350	63.84	2	0.91	5.7	1.13	25.7	0.76	33.6
AIVAGDQNVEYK	Y117	Isoform 3 of Four and a half LIM domains protein 1	FHL1	FHL1	Q13642-3	Y11(Phospho)	214	54		2	922.982	56.92	4	2.86	25.8	7.27	38.9	7.01	29.7
DGSLASNPYSGDLTK	Y858	Isoform 4 of Ubiquitin-associated protein 2-like	UBAP2L	UBP2L	Q14157-4	Y9(Phospho)	227	46		3	688.341	59.43	2	9.92	52.9	16.12	39.7	12.24	20.8
SAVGFEYQGK	Y141	Isoform 1 of Src substrate cortactin	CTTN	SRC8	Q14247-1	Y7(Phospho)	152	43		2	812.414	57.71	3	8.95	6.9	19.98	57.2	16.13	10.2
LPSSPVYEDAASFK	S381&Y384	Isoform 1 of Src substrate cortactin	CTTN	SRC8	Q14247-1	S4(Phospho); Y7(Phospho)	214	43		2	1065.006	70.32	3	8.10	0.4	18.52	62.8	14.18	36.9
GPVSGTEPEPVYSMEAADYR	Y416	Isoform 1 of Src substrate cortactin	CTTN	SRC8	Q14247-1	Y19(Phospho)	219	53		2	1232.550	64.60	3	3.03	96.7	5.25	19.4	4.65	53.1
TQTPPVSPAPQPTEERLPSSPVYEDAASFK	S418&Y421	Isoform 1 of Src substrate cortactin	CTTN	SRC8	Q14247-1	S19(Phospho); Y23(Phospho)	184	53		3	1282.287	67.45	2	5.63	61.4	22.46	29.9	15.02	6.6
LPSSPVYEDAASFK	Y421	Isoform 1 of Src substrate cortactin	CTTN	SRC8	Q14247-1	Y7(Phospho)	249	60		3	683.684	69.64	4	6.60	36.8	18.12	37.6	15.11	23.5
GPVSGTEPEPVYSMEAADYR	Y409&Y416	Isoform 1 of Src substrate cortactin	CTTN	SRC8	Q14247-1	Y12(Phospho); Y19(Phospho)	284	51		3	854.023	52.96	2	1.51	50.8	2.93	72.9	3.74	120.9
NASTFEDVTQVSSAYQK	Y297	Isoform 1 of Src substrate cortactin	CTTN	SRC8	Q14247-1	Y15(Phospho)	286	56		4	604.047	64.69	3	2.44	0.8	6.44	14.9	6.32	32.7
SAVGFDYQGK	Y178	Isoform 1 of Src substrate cortactin	CTTN	SRC8	Q14247-1	Y7(Phospho)	n/a	29		3	537.272	57.49	1	4.87		13.89		13.81	
DGYQQNFK	Y670	Isoform 2 of Caprin-1	CAPRIN1	CAPR1	Q14444-2	Y3(Phospho)	n/a	22		2	769.376	48.65	1	4.25		8.45		8.44	
AYSSFGGGR	Y12	Isoform Short of Eukaryotic translation initiation factor 4H	EIF4H	IF4H	Q15056-2	Y2(Phospho)	126	29		2	605.777	46.41	3	19.69	45.7	23.49	14.9	15.87	0.8
AAPFSLEYR	Y17	Inorganic pyrophosphatase	PPA1	IPYR	Q15181	Y8(Phospho)	138	39		2	681.837	66.98	3	10.88	70.4	22.87	6.7	13.92	4.1
VIEDDPEAVYTTTGGK	Y791	Isoform 2 of Ephrin type-A receptor 7	EPHA7	EPHA7	Q15375-2	Y10(Phospho)	221	65	3.55	2	1117.057	63.53	4	0.96	6.5	1.12	9.3	0.98	13.2
ADQEGDEELYFHFK	Y597	Isoform 2 of Ephrin type-A receptor 7	EPHA7	EPHA7	Q15375-2	Y10(Phospho)	254	52		3	756.023	71.96	2	0.96	8.6	1.33	5.1	1.26	1.1
VNFCPLPSEQCYQAPGGPEDR	Y55	Thyroid receptor-interacting protein 6	TRIP6	TRIP6	Q15654	Y12(Phospho)	162	38		3	910.734	66.71	1	3.05		5.64		5.02	
HTDDEMTGYVATR	Y182	Isoform Mxi2 of Mitogen-activated protein kinase 14	MAPK14	MK14	Q16539-3	Y9(Phospho)	231	50		3	602.264	46.17	4	1.01	15.1	1.14	14.8	0.78	20.8
EAEYAEAR	Y95	Isoform 2 of SUZ domain-containing protein 1	SZRD1	SZRD1	Q7Z422-2	Y4(Phospho)	102	28		2	624.279	38.55	1	9.38		15.84		18.98	

Supplementary Table 2

peptide - sequence	phospho-site	protein descriptions	gene name	synonyms	swissprot protein group accessions	phosphorylation modification	phosphoRS Binomial Peptide Score	Mascot Ion Score	SequestXCorr	peptide charge	m/z [Th]	retention time (min)	# PSM	30 min 127/126 PP/Kin^{-}	CV (%)	90 min 129/128 PP/Kin^{-}	CV (%)	150 min 131/130 PP/Kin^{-}	CV (%)
**phosphotyrosine peptides**																			
AADQFDIYSSQQSK	Y35	Isoform 2 of UPF0461 protein C5orf24	C5orf24	CE024	Q7Z6I8-2	Y8(Phospho)	237	35		3	709.346	60.64	1	5.87		9.86		12.47	
IELCAYSCEDR	Y599	Isoform 2 of NEDD4-binding protein 2	N4BP2	N4BP2	Q86UW6-2	Y6(Phospho)	156	33		2	862.865	61.35	1	1.19		1.78		1.47	
ATTPPNQGRPDSPVYANLQELK	T231;S240&Y243	Isoform 3 of Rho GTPase-activating protein 12	ARHGAP12	RHG12	Q8IWW6-3	T3(Phospho); S12(Phospho); Y15(Phospho)	149	34		3	1032.155	70.51	1	0.92		1.25		1.22	
ATTPPNQGRPDSPVYANLQELK	S240&Y243	Isoform 3 of Rho GTPase-activating protein 12	ARHGAP12	RHG12	Q8IWW6-3	S12(Phospho); Y15(Phospho)	117	39	4.05	4	754.377	68.28	2	1.46	29.2	1.95	30.8	1.92	39.1
TLEPVKPPTVPNDYMTSPAR	Y213	Isoform 11 of Abl interactor 1	ABI1	ABI1	Q8IZP0-11	Y14(Phospho)	136	46		3	923.143	63.23	4	1.48	1.2	1.83	5.8	1.57	8.4
AEIYALNR	Y40	Coiled-coil domain-containing protein 23	CCDC23	CCD23	Q8N300	Y4(Phospho)	192	37		2	629.823	59.55	1	7.12		20.18		13.93	
NRPPFGQGYTQPGPGYR	Y392&Y399	Isoform 2 of Protein TFG	TFG	TFG	Q92734-2	Y9(Phospho); Y16(Phospho)	202	33		3	761.011	51.59	2	7.26	58.2	14.75	72.0	12.05	38.4
NRPPFGQGYTQPGPGYR	Y392	Isoform 2 of Protein TFG	TFG	TFG	Q92734-2	Y16(Phospho)	179	26		3	734.355	57.74	2	6.37	48.6	7.75	39.6	6.35	31.5
EAIIPGSVYDR	Y958	Isoform 2 of Regulator of nonsense transcripts 1	UPF1	RENT1	Q92900-2	Y9(Phospho)	137	32		2	764.883	62.78	1	4.43		9.35		11.01	
IGGDAGTSLNSNDYGYGGQK	Y60	Isoform 1 of Far upstream element-binding protein 1	FUBP1	FUBP1	Q96AE4-1	Y14(Phospho)	238	59		3	838.064	60.77	3	3.36	105.6	7.62	16.9	11.13	91.2
EYAEDDNIYQQK	Y68	Isoform 1 of Ubiquitin thioesterase OTUB1	OTUB1	OTUB1	Q96FW1-1	Y9(Phospho)	n/a	32	3.90	3	685.321	53.24	1	3.27		6.63		8.34	
HGDEIYIAPSGVQK	Y57	Isoform 1 of Methylthioribulose-1-phosphate dehydratase	APIP	MTNB	Q96GX9-1	Y6(Phospho)	n/a	32		3	684.691	61.51	1	1.93		13.85		11.35	
AVLYADYR	Y638	Isoform 3 of Kin of IRRE-like protein 1	KIRREL	KIRR1	Q96J84-3	Y4(Phospho)	86	27		2	640.318	60.20	1	1.11		1.56		1.55	
LAELSDYR	Y614	Isoform 1 of RNA-binding protein 14	RBM14	RBM14	Q96PK6-1	Y7(Phospho)	n/a	29		2	638.313	63.59	1	3.63		7.10		7.45	
VADLTEQYNEQYGAVR	Y194	Isoform 5 of RNA-binding protein 14	RBM14	RBM14	Q96PK6-5	Y12(Phospho)	163	73		2	1083.009	64.25	1	8.48		10.45		7.21	
LGEYEDVSR	Y98	Tubulin-folding cofactor B	TBCB	TBCB	Q99426	Y4(Phospho)	99	39		2	688.819	52.45	1	3.97		7.15		7.20	
VKEEGYELPYNPATDDYAVPPPR	Y398&Y409	Isoform 3 of Docking protein 1	DOK1	DOK1	Q99704-3	Y6(Phospho); Y17(Phospho)	190	45		3	1080.177	65.56	1	3.82		11.31		13.39	
SAEEAPLYSK	Y389	Isoform 2 of Tyrosine-protein phosphatase non-receptor type 18	PTPN18	PTN18	Q99952-2	Y8(Phospho)	n/a	41	3.42	2	816.919	53.23	1	4.26		8.60		9.45	
GNEYQPSNIK	Y53	39S ribosomal protein L34, mitochondrial	MRPL34	RM34	Q9BQ48	Y4(Phospho)	n/a	31	3.40	2	844.427	46.98	1	8.03		11.84		14.90	
EGYVPQEEVPVYENK	Y45	Isoform 2 of Partner of Y14 and mago	WIBG	WIBG	Q9BRP8-2	Y12(Phospho)	235	42		2	1159.572	62.37	2	9.67	35.9	18.07	70.0	16.48	24.5
EEIQDEEDDDDYVEEGEEEEEEEGGLRGEK	Y235	Isoform 3 of Acidic leucine-rich nuclear phosphoprotein 32 family member E	ANP32E	AN32E	Q9BTT0-3	Y12(Phospho)	174	45		3	1399.580	68.77	1	1.11		0.73		0.88	
LSPADDELYQR	Y42	Isoform a of NADH dehydrogenase [ubiquinone] 1 alpha subcomplex assembly factor 3	NDUFAF3	NDUF3	Q9BU61-1	Y9(Phospho)	153	48		2	808.383	60.05	1	9.38		21.13		14.42	
QNQFYDTQVIK	Y111	Isoform 3 of Heterogeneous nuclear ribonucleoprotein U-like protein 1	HNRNPUL1	HNRL1	Q9BUJ2-3	Y5(Phospho)	197	32		2	961.495	63.59	2	4.09	115.5	6.49	62.8	4.98	88.8
VDELSLYSVPEGQSK	Y43	Isoform 2 of Apolipoprotein O	APOO	APOO	Q9BUR5-2	Y7(Phospho)	n/a	33		3	730.377	68.05	1	4.03		10.45		13.28	
SIADSEESEAYK	Y278	UPF0549 protein C20orf43	C20orf43	CT043	Q9BY42	Y11(Phospho)	184	75		2	933.944	51.39	1	3.12		9.35		7.12	
VATAYRPPALR	Y446	Isoform 2 of Eukaryotic translation initiation factor 2A	EIF2A	EIF2A	Q9BY44-2	Y5(Phospho)	145	27		3	508.615	51.89	1	14.68		21.20		16.54	
QIYNPPSGK	Y481	Isoform 1 of Apoptosis inhibitor 5	API5	API5	Q9BZZ5-1	Y3(Phospho)	157	34		2	771.411	46.95	3	7.38	16.2	11.24	22.2	13.03	13.6
VVDYSQFQESDDADEDYGR	Y26	Isoform 1 of Nuclear ubiquitous casein and cyclin-dependent kinase substrate 1	NUCKS1	NUCKS	Q9H1E3-1	Y17(Phospho)	233	69		2	1274.025	62.89	4	1.37	48.1	1.45	83.6	1.73	122.4
VVDYSQFQESDDADEDYGR	S19&Y26	Isoform 1 of Nuclear ubiquitous casein and cyclin-dependent kinase substrate 1	NUCKS1	NUCKS	Q9H1E3-1	S10(Phospho); Y17(Phospho)	159	51		2	1314.008	63.25	2	1.09	49.2	1.44	81.8	1.69	71.0
VVDYSQFQESDDADEDYGRDSGPPTK	S19;Y26&S30	Isoform 1 of Nuclear ubiquitous casein and cyclin-dependent kinase substrate 1	NUCKS1	NUCKS	Q9H1E3-1	S10(Phospho); Y17(Phospho); S21(Phospho)	143	50		4	905.372	63.48	2	0.82	11.8	1.08	15.0	0.80	30.6
VVDHYENPR	Y43	Isoform 1 of Iron-sulfur cluster assembly enzyme ISCU, mitochondrial	ISCU	ISCU	Q9H1K1-1	Y5(Phospho)	120	28		2	719.340	42.35	1	4.16		6.45		9.16	
TVCSTYLQSR	Y359	Isoform 2 of Homeodomain-interacting protein kinase 3	HIPK3	HIPK3	Q9H422-2	Y6(Phospho)	n/a	43	3.58	2	762.360	49.16	1	0.89		1.05		0.85	
VILATENDYCK	Y148	Isoform 2 of Zinc finger matrin-type protein 3	ZMAT3	ZMAT3	Q9HA38-2	Y9(Phospho)	163	45		2	932.469	62.80	1	6.48		9.05		8.63	
VPSEGAYDIILPR	Y399	Isoform 1 of G-protein coupled receptor family C group 5 member C	GPRC5C	GPC5C	Q9NQ84-1	Y7(Phospho)	209	59		3	580.305	72.78	2	1.11	6.7	1.33	7.1	1.49	46.0
NEEENIYSVPHDSTQGK	Y1105	Rho GTPase-activating protein 35	ARHGAP35	RHG35	Q9NRY4	Y7(Phospho)	n/a	37		3	829.061	52.72	1	1.58		3.08		4.30	
AEFAEYASVDR	Y227	Phosphoprotein associated with glycosphingolipid-enriched microdomains 1	PAG1	PHAG1	Q9NWQ8	Y6(Phospho); S8(Phospho)	122	46		2	823.839	58.64	1	0.93		1.29		1.33	
SGQSLTVPESTYTSIQGDPQR	Y341	Phosphoprotein associated with glycosphingolipid-enriched microdomains 1	PAG1	PHAG1	Q9NWQ8	Y12(Phospho)	225	67		2	1280.610	60.91	2	1.06	29.6	1.09	46.7	0.98	44.3
AEFAEYASVDRNK	Y227&S229	Phosphoprotein associated with glycosphingolipid-enriched microdomains 1	PAG1	PHAG1	Q9NWQ8	Y6(Phospho); S8(Phospho)	177	33	3.96	3	706.662	59.77	1	0.89		1.43		0.99	
SVDGDQGLGMEGPYEVLK	Y163	Phosphoprotein associated with glycosphingolipid-enriched microdomains 1	PAG1	PHAG1	Q9NWQ8	Y14(Phospho)	254	30		3	811.404	69.87	1	1.72		2.78		2.57	
ENDYESISDLQQGR	Y417	Phosphoprotein associated with glycosphingolipid-enriched microdomains 1	PAG1	PHAG1	Q9NWQ8	Y4(Phospho)	245	42		3	654.961	60.88	1	1.35		2.61		1.98	
AEFAEYASVDR	Y227	Phosphoprotein associated with glycosphingolipid-enriched microdomains 1	PAG1	PHAG1	Q9NWQ8	Y6(Phospho)	212	43		3	522.906	59.86	4	1.07	17.5	1.14	7.8	1.19	27.4
DSSSQENMVEDCLYETVK	Y181	Phosphoprotein associated with glycosphingolipid-enriched microdomains 1	PAG1	PHAG1	Q9NWQ8	Y14(Phospho)	n/a		4.48	3	896.733	70.46	1	3.60		5.51		7.29	
TPNSTLPPAGRPSEEPEPDYEAIQTLNR	Y387	Phosphoprotein associated with glycosphingolipid-enriched microdomains 1	PAG1	PHAG1	Q9NWQ8	Y20(Phospho)	n/a		4.50	3	1130.213	65.77	1	2.64		5.01		3.76	
NITYEELR	Y199	Isoform 1 of OCIA domain-containing protein 1	OCIAD1	OCAD1	Q9NX40-1	Y4(Phospho)	n/a	32		2	673.832	59.96	1	3.66		9.50		9.39	
EDAANNYAR	Y103	Isoform 2 of Tubulin alpha-8 chain	TUBA8	TBA8	Q9NY65-2	Y7(Phospho)	137	30		2	666.793	36.88	1	5.78		7.19		5.58	
VGINYQPPTVVPGGDLAK	Y357	Isoform 2 of Tubulin alpha-8 chain	TUBA8	TBA8	Q9NY65-2	Y5(Phospho)	n/a		3.71	3	788.431	68.58	1	2.61		3.99		3.01	
REEPEALYAAVNK	Y968	Isoform 4 of Intersectin-2	ITSN2	ITSN2	Q9NZM3-4	Y8(Phospho)	263	43		3	676.690	62.47	3	1.27	2.7	2.28	21.1	2.61	16.3
SPDEAYAIAK	Y84	Isoform 2 of Succinyl-CoA ligase [ADP-forming] subunit beta, mitochondrial	SUCLA2	SUCB1	Q9P2R7-2	Y6(Phospho)	162	38		2	801.913	56.38	2	1.95	23.6	2.89	39.8	2.83	31.1
ANQEQYAEGK	Y123	Isoform 2 of Mortality factor 4-like protein 1	MORF4L1	MO4L1	Q9UBU8-2	Y6(Phospho)	138	43		2	838.408	42.08	3	5.07	190.1	16.98	117.8	9.80	57.3
VSLEEIYSGCTK	Y176	DnaJ homolog subfamily B member 4	DNAJB4	DNJB4	Q9UDY4	Y7(Phospho)	198	36		3	641.990	66.48	1	2.79		7.55		7.74	
EKQPVAGSEGAQYR	Y145	Isoform 2 of Testin	TES	TES	Q9UGI8-2	Y13(Phospho)	189	32	3.29	3	686.685	41.52	1	3.79		7.12		12.95	
EGDPAIYAER	Y251	Isoform 2 of Testin	TES	TES	Q9UGI8-2	Y7(Phospho)	n/a	28		2	715.332	49.07	1	3.21		9.59		7.36	
LQLDNQYAVLENQK	Y244	Isoform 1 of Protein CDV3 homolog	CDV3	CDV3	Q9UKY7-1	Y7(Phospho)	253	68	4.39	2	1107.583	67.69	4	13.16	39.6	26.53	23.2	13.43	40.6
TPQGPPEIYSDTQFPSLQSTAK	Y190	Isoform 1 of Protein CDV3 homolog	CDV3	CDV3	Q9UKY7-1	Y9(Phospho)	210	67		3	977.492	70.40	2	2.67	60.7	17.07	128.7	10.70	118.2
EVDYSGLR	Y95	Isoform 1 of Protein CDV3 homolog	CDV3	CDV3	Q9UKY7-1	Y4(Phospho)	n/a	37		2	624.297	50.96	1	3.07		6.79		8.09	
DPNSPLYSVK	Y89	Isoform 2 of ATP-dependent RNA helicase DDX19B	DDX19B	DD19B	Q9UMR2-2	Y7(Phospho)	n/a	31		2	829.434	62.26	1	2.69		5.55		5.17	
LGAAPEEESAYVAGEK	Y169	Isoform 4 of NSFL1 cofactor p47	NSFL1C	NSF1C	Q9UNZ2-6	Y11(Phospho)	n/a	29		3	720.361	62.10	1	2.42		3.11		3.61	
DSMSAAEVGTGQYATTK	Y453	Isoform C of Band 4.1-like protein 3	EPB41L3	E41L3	Q9Y2J2-3	Y13(Phospho)	242	36		3	757.693	47.54	1	1.37		2.21		1.64	
HLDTLTEHYDIPK	Y103	Isoform 3 of Protein FAM32A	FAM32A	FA32A	Q9Y421-3	Y9(Phospho)	250	36	3.29	3	707.366	68.62	7	10.59	40.1	15.72	31.8	12.07	2.3
ASSPAESSPEDSGYMR	Y742	Insulin receptor substrate 2	IRS2	IRS2	Q9Y4H2	Y14(Phospho)	107	45		2	998.414	38.71	1	0.84		1.35		1.06	
APYTCGGDSDQYVLMSSPVGR	Y823	Insulin receptor substrate 2	IRS2	IRS2	Q9Y4H2	Y12(Phospho)	249	50		3	862.380	62.63	1	1.94		4.45		4.87	
AALIYTCTVCR	Y39	Zinc finger protein 706	ZNF706	ZN706	Q9Y5V0	Y5(Phospho)	210	47		2	818.893	61.95	2	7.97	122.6	27.43	0.4	14.38	3.4

Supplementary Table 2
